# Plant traits and associated ecological data from Afromontane grasslands of Maloti-Drakensberg, South Africa

**DOI:** 10.1038/s41597-025-06045-x

**Published:** 2025-11-12

**Authors:** Aud H. Halbritter, Vigdis Vandvik, Nicole N. Bison, Vincent Ralph Clark, Marcella Cross, Michelle Greve, Julia Kemppinen, Nicola Kühn, Brian S. Maitner, Sean T. Michaletz, Jocelyn Navarro, Pekka Niittynen, Peter Christiaan le Roux, Joseph D. M. White, Bezawit Yilma Abebe, Nadine Michaela Arzt, Ludwig Baldaszti, Lina Aragón, Samuel Beale, Kristine Birkeli, Anya P. Courtenay, Hilary Rose Dawson, Liliya Kirilova Draganova, Lauren E. Gillespie, Kaleb A. Goff, Anna Tariro Deirdre Gowera, Axel Gualdoni-Becerra, Onalenna Gwate, Liyenne Wu Chen Hagenberg, Priya Hansda, Rebecca Harris, Joshua Lee, Michelle A. Louw, Aino-Maja Määttänen, Nathan Malamud, Lesego Malekana, Bridgette Mc Millan, Nasser Rabi, Michael Mustri, Samson Mekasha, Sara Shemsu Nasir, Francisco Navarro-Rosales, Bismark Ofosu-Bamfo, Akuonani Zakeyo Phiri, Eline Sterre Rentier, Carmen Vázquez-Ribera, Grecia Rivas, Tin Widyani Satriawan, Imke Chrissie Smit, Paul Abayomi Sobowale Soremi, Ragnhild Svensen Stokka, Jiří Šubrt, Jonas Trepel, Jakub D. Wieczorkowski, Brian J. Enquist

**Affiliations:** 1 Department of Biological Sciences, University of Bergen, Bergen, Norway; 2 Bjerknes Centre for Climate Research, University of Bergen, Bergen, Norway; 3 Department of Botany, The University of British Columbia, Vancouver, Canada; 4 Biodiversity Research Centre, The University of British Columbia, Vancouver, Canada; 5 Afromontane Research Unit & Department of Geography, University of the Free State: Qwaqwa Campus, Phuthaditjhaba, South Africa; 6 Department of Plant and Soil Sciences, University of Pretoria, Hatfield, South Africa; 7 Botany and Mycology Unit, Finnish Museum of Natural History, University of Helsinki, Helsinki, Finland; 8 Organismal and Evolutionary Biology Research Programme, Faculty of Biological and Environmental Sciences, University of Helsinki, Helsinki, Finland; 9 Jodrell Laboratory, Royal Botanic Gardens Kew, Richmond, UK; 10 Department of Integrative Biology, University of South Florida, St. Petersburg, USA; 11 Department of Ecology and Evolutionary Biology, University of Arizona, Arizona, USA; 12 University of Jyväskylä, Jyväskylä, Finland; 13 Addis Ababa University, Addis Ababa, Ethiopia; 14 School of GeoSciences, The University of Edinburgh, Edinburgh, UK; 15 Taxonomy and Macroecology, Royal Botanic Garden Edinburgh, Edinburgh, UK; 16 Department of Biology, University of Miami, Coral Gables, USA; 17 La Trobe University, Melbourne, Australia; 18 Taxonomy and Macroecology, Royal Botanic Garden Edinburgh, Edinburgh, EH3 5LR UK; 19 Institute of Ecology and Evolution, Department of Biology, University of Oregon, Eugene, USA; 20 Research School of Biology, Australian National University, Canberra, Australia; 21 University of Bergen, Department of Biological Sciences, Bergen, Norway; 22 Department of Computer Science, Stanford University, Standford, USA; 23 Department of Plant and Microbial Biology, North Carolina State University, Raleigh, USA; 24 School of Animal, Plant and Environmental Sciences/ Wits Mining Institute, University of the Witwatersrand, Johannesburg, South Africa; 25 SATCAP Research Centre, Wits Mining Institute, University of the Witwatersrand, Johannesburg, South Africa; 26 University of Göttingen, Department of Biodiversity, Macroecology & Biogeography, Göttingen, Germany; 27 Department of Ecology and Environmental Sciences, Umeå University, Umeå, Sweden; 28 Plant Ecology Laboratory, School of Environmental Sciences, Jawaharlal Nehru University, New Delhi, India; 29 Department of Forest and Rangeland Stewardship, Colorado State University, Fort Collins, USA; 30 Hawkesbury Institute for the Environment, Western Sydney University, Sydney, Australia; 31 Copernicus Institute of Sustainable Development, Utrecht University, Utrecht, The Netherlands; 32 Geography Research Unit, University of Oulu, Oulu, Finland; 33 Finnish Environment Institute, Helsinki, Finland; 34 Department of Botany, University of British Columbia, Vancouver, Canada; 35 Biodiversity Research Centre, 2212 Main Mall, Vancouver, Canada; 36 W.K Kellogg Biological Station, Michigan State University, Hickory Corners, USA; 37 Department of Plant Biology and Biodiversity Management, College of Natural and Computational Sciences, Addis Ababa University, Addis Ababa, Ethiopia; 38 Department of Biology, University of Oxford, Oxford, United Kingdom; 39 Department of Biological Sciences, School of Sciences, University of Energy and Natural Resources, Sunyani, Ghana; 40 National Autonomous University of Mexico, Mexico City, Mexico; 41 Centro de Investigación en Alimentación y Desarrollo, A.C., Hermosillo, México; 42 Department of Geography, National University of Singapore, Singapore, Singapore; 43 Federal University of Agriculture, Abeokuta, Nigeria; 44 Center for Ecological Dynamics in a Novel Biosphere (ECONOVO), Department of Biology, Aarhus University, Aarhus, Denmark; 45 School of GeoSciences, The University of Edinburgh, Edinburgh, Scotland; 46 Taxonomy and Macroecology, Royal Botanic Garden Edinburgh, Edinburgh, Scotland; 47 The Santa Fe Institute, Santa Fe, USA

**Keywords:** Biodiversity, Grassland ecology, Climate-change ecology, Ecophysiology, Ecosystem ecology

## Abstract

The Afromontane region harbors ancient grasslands with high levels of endemism, now under threat from land-use change, biological invasions and encroachment, and climate warming. As part of an international Plant Functional Traits Course we collected comprehensive trait data in five sites along an elevation gradient from 2,000–2,800 m a.s.l. and in a climate warming experiment at 3,064 m a.s.l. in the Maloti-Drakensberg, South Africa. We sampled 24,405 aboveground and 94 root trait measurements from 171 vascular plant taxa paired with 11 other datasets reflecting vegetation and structure, leaf and ecosystem carbon and water fluxes, leaf hyperspectral reflectance, and microclimatic and environmental data. Our data provide the first recorded trait data for 47 vascular plant species and more than double the trait data coverage from the Maloti-Drakensberg (106% increase). This study offers insights into plant and ecosystem functioning, provides a baseline for assessing impacts of environmental change, builds local competence, and aligns with similar data from China, Svalbard, Peru, and Norway.

## Background & Summary

The Afromontane region supports a global biodiversity hotspot with high levels of endemism^1,2^. The biodiversity and ecosystems of this region have been shaped by the interplay between climate, fire, grazing, and topography for millennia. In particular, fire, frost, and high evapotranspiration has promoted grasslands as a dominant vegetation type across the Afromontane region, and these factors have also shaped the vegetation structure, composition, diversity, and functional ecology of these grasslands^3,4^. Across the southern African Afromontane mountain ranges, including for the Maloti-Drakensberg, grassland species make up the majority of plant endemics (70–90%)^5,6^. In contrast, diversity and endemism is lower in the Afrotemperate forests of these mountains, which are generally small and restricted to topographic fire refugia^7^. Afromontane ecosystems and in particular grasslands also support important ecosystem services such as erosion control, grazing resources, and water regulation, benefiting both local communities and more distant populations^8–10^.

African mountain ecosystems have served as biodiversity refugia during periods of past climate variations^6,11^, but are now under threat from the compounding effects of biological invasions, land-use change, and climate change^12–15^. A shift from traditional low-intensity free-range grazing by diverse assemblages of domestic and wild herbivores to higher densities of livestock, often in fenced-off parcels, leads to overgrazing and loss of native diversity^14,16,17^. Fire regimes have also been altered, with fire suppression in some areas more frequent burning in others^16^. These changes to grazing and fire regimes facilitate encroachment by both native and invasive alien woody plants^15,18^. These threats are compounded by climate warming^19^, resulting in further loss of biodiversity and ecosystem functioning^20,21^. Despite these challenges, much of the Maloti-Drakensberg remains under limited formal protection (79% is communally owned rangeland^22^).

Functional trait-based approaches provide a comprehensive framework for understanding plant community and ecosystem responses to environmental variation and change^23–25^. Functional traits can link how changes in organismal physiology, morphology, and life history impact ecosystem functioning and offer insights into how species and communities respond to shifting environmental drivers^25,26^. Data on intraspecific trait variation can inform individual and population-level responses and effects, including response to shifting selection pressures^27–29^. Integrating trait data with physiological measurements, ecosystem fluxes, and environmental variables allows researchers to investigate biodiversity assembly and ecosystem functioning across scales, from individuals to landscapes^30,31^.

**Fig. 1:**
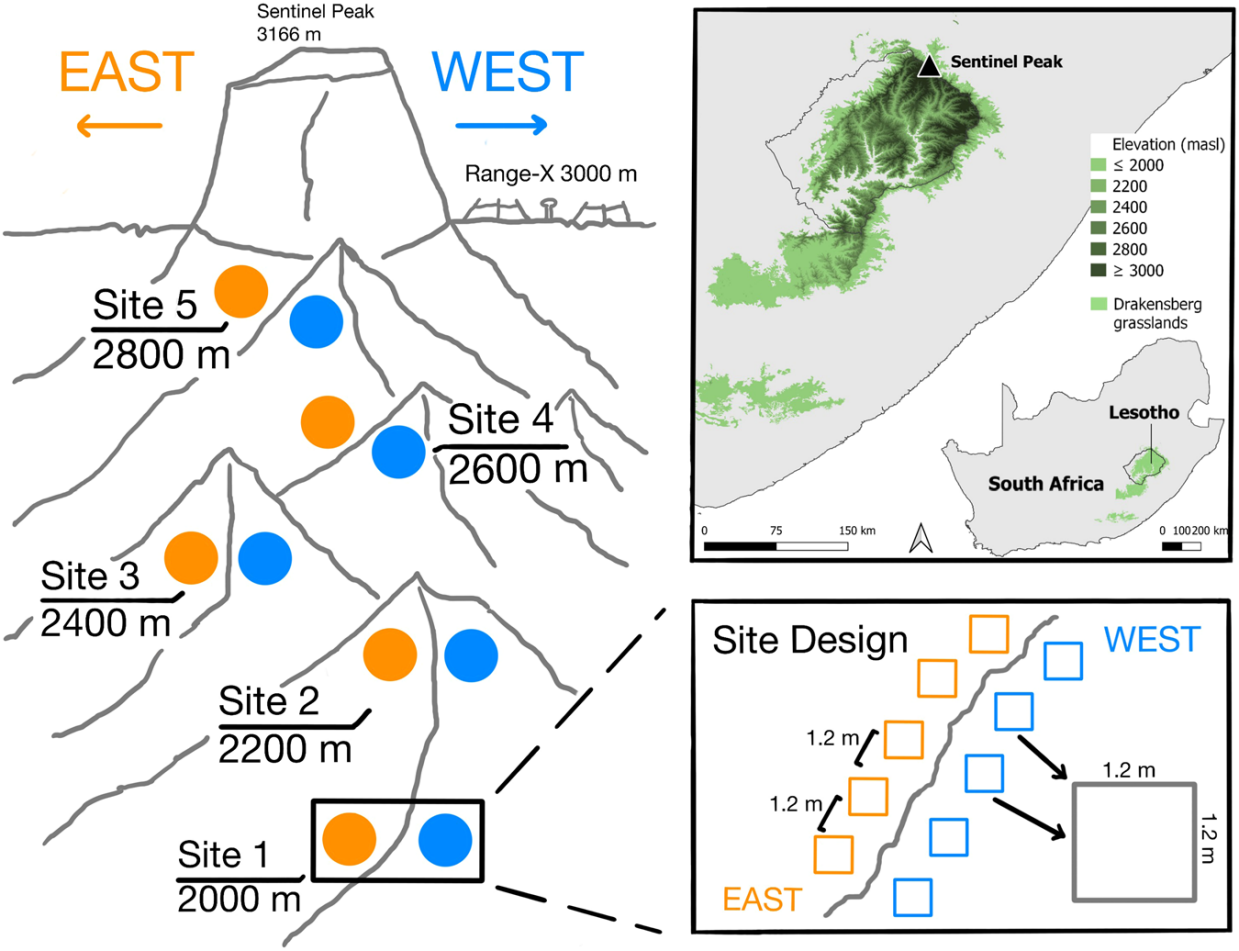
Conceptual figure of the study design and map of the site location in the Afromontane grasslands of the Maloti-Drakensberg escarpment. The study was carried out in the Free State and KwaZulu-Natal Provinces, South Africa. The study area follows an elevation gradient with five sites from 2,000-2,800 meters above sea level (m a.s.l), with five 1.2 × 1.2 m plots each on the east- and west-facing aspects of the ridge per elevation. At the 3,064 m a.s.l. RangeX site, we sampled from five 1 × 1 m plots in extant climate and five in experimentally warmed plots inside OTCs. The inset map shows the elevation profile of the Maloti-Drakensberg grasslands with the study site indicated, and the location of the Maloti-Drakensberg within South Africa^159,160^.

Here, we report on the first comprehensive sampling of plant functional traits and associated biotic and abiotic variables from the Afromontane grasslands of South Africa. In 2023, we selected five sites along an 800 m elevation gradient in the northern Maloti-Drakensberg from 2,000 to 2,800 m above sea level (a.s.l.) where we set up sampling plots on east- and west-facing slopes, and we also sampled an existing climate warming experiment at 3,064 m a.s.l.. (Fig. 1). From this study system, we collected aboveground and root plant functional trait data along with 11 associated datasets on vegetation composition, structure, and biomass, leaf assimilation-temperature responses, leaf hyperspectral reflectance, vegetation thermal imagery, ecosystem CO_2_ and H_2_O fluxes, microclimate, and environmental variables. As full coverage of all taxa, sites, and plots was not possible for all approaches, sampling designs for each dataset was selected to facilitate linkages to the functional trait and other data along the elevation gradient.

This paper reports on 1,038 plant observations and 24,405 aboveground trait measurements from 171 flowering plant taxa (Table 1). This more than doubles the number of unique trait measurements available from the Maloti-Drakensberg flora, relative to the TRY database^32^, and provides the first published records of functional trait data for 47 species in the regional flora. We further report on 94 belowground trait measurements from 5 focal taxa, 96 leaf assimilation-temperature responses from 9 focal taxa, 1,089 leaf hyperspectral reflectance from 56 focal taxa, 392 plot-scale ecosystem flux measurements, along with data on above- and belowground community structure, biomass, microclimate, environment, and soils (see Table 1).

**Table 1:** Description and location of the plant functional trait and associated datasets from an elevation gradient and a warming experiment in the Maloti-Drakensberg, South Africa, in 2023.

Dataset	Response variable	Study system	Number of observations	Number of taxa	Number of plots*	Aspect	Sites	Citation for raw data, clean data and code
i	Plant species composition	gradient experiment	1,038 156	150 39	50 10	both east	5 1	Raw and clean data^146^, code^149^
ii	Vegetation height and structure	gradient experiment	250 50	— —	50 10	both east	5 1	Raw and clean data^146^, code^149^
iii	Aboveground biomass	gradient	56	—	50	both	5	Raw and clean data^146^, code^149^
iv-a iv-b	Aboveground traits Aboveground traits	gradient experiment	21,921 2,484	154 41	50 10	both east	5 1	Raw and clean data^146^, code^149^
v	Root traits	gradient	94	5	—	west	4	Raw and clean data^146^, code^154^
vi-a vi-b	Root biomass validation Ground penetrating radar mapping	gradient gradient	720 3,055	— —	80^a^ 40	west both	4 4	Raw and clean data^146^, code^154^
vii	Leaf assimilation-temperature responses	gradient	96	9	—	both	5	Raw data^146^, clean data^147^, code^147^
viii	Leaf hyperspectral reflectance	gradient	1,089	56	50	both	5	Raw and clean data^146^, code^155^
ix	Vegetation thermal imagery (FLIR camera)	gradient	8	4	—	both	2	Raw and clean data^148^, code^156^
x	Ecosystem CO_2_ fluxes Ecosystem H_2_O fluxes	gradient gradient	194 198	— —	50 50	both	5	Raw and clean data^146^, code^157^
xi	Microclimate	gradient experiment	2,558,857 1,457,145	—	10 60	both east	5 1	Raw and clean data^146^, code^157^
xii	Soil texture and nutrients	gradient	444	—	50	both	5	Raw and clean data^146^, code^154^
xiii	Geodiversity and microtopography	gradient	550	—	50	both	5	Raw and clean data^146^, code^149^

This table summarizes information on dataset numbers, response variable(s), study system (elevation gradient, warming experiment, or combined data), number of observations, taxa, plots, aspect, site, and location of the primary data, the final published data, and the code for extracting and cleaning data from the primary data. *the number of sampling plots within the elevation gradient and/or warming experiment from which measurements are taken, with the exception of dataset vi, where separate transects were set up at the relevant sites and aspects to avoid destructive sampling of plots. ^a^refers to the number of plots along these additional transects.

By making these data available, we aim to contribute towards a better ecological understanding of Afromontane grassland ecosystems and facilitate research on the Maloti-Drakensberg socio-ecological system. The dataset provides a baseline for future studies and a valuable resource for understanding how environmental gradients and a warming climate influence plant traits, ecosystem processes, and biodiversity assembly. Our data were collected by the Plant Functional Traits Course 7 (PFTC7), as part of an international course series for students specializing in the theory and methods of trait-based ecology, see also^33,34^. The data presented in this data paper align with the data collected from similar courses conducted in China^35^, Svalbard^36^, Peru^37^, and Norway^38^, paving the way for comparative research and future studies^39^, collaborations, and networking. The PFTC7 provided opportunities for local capacity building, contributing to a regional community of practice for southern African mountain research, conservation, and management, including to the Mont-aux-Sources Long-term Social-Ecological Research Site (MaS LTSER) in the northern Maloti-Drakensberg^40^, the Maloti-Drakensberg Transfrontier Programme, and local conservation and livelihood creation initiatives such as the proposed indigenous community-led Witsieshoek Community Conservation Area.

## Methods

### Data management and workflows

**Fig. 2:**
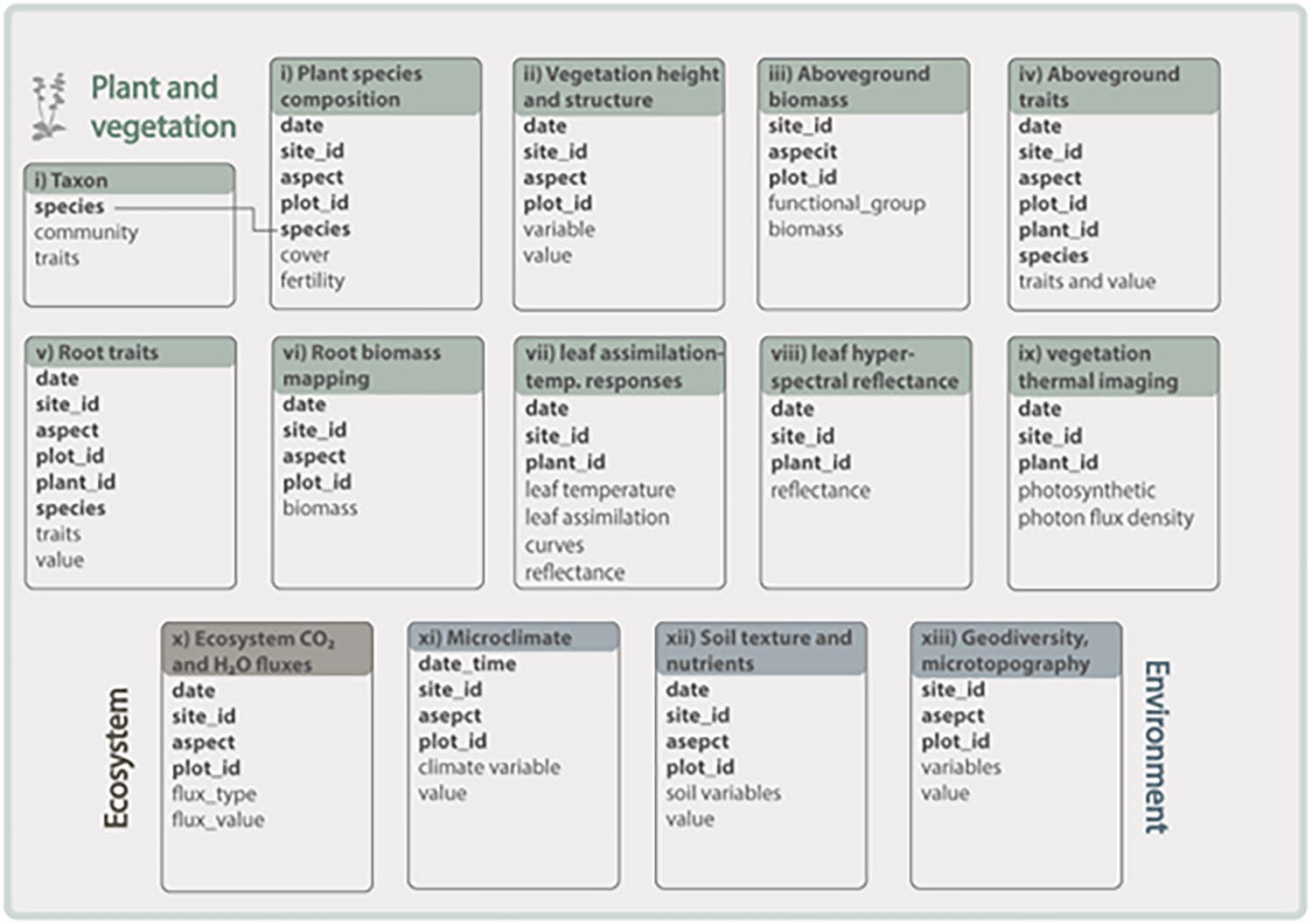
Data structure for the PFTC7 traits campaign. Boxes represent individual data tables for plant and vegetation data (green), ecosystem data (brown), and environmental data (blue). For each data table, the number and name of the dataset is given in the colored title area and examples of variables are listed inside the box. Bold variable names indicate shared variables that can function as keys to join datasets (see Table 2), normal font variable names indicate (examples of) unique variables for that dataset. The lines linking boxes (i) and (ii) exemplify links using **species** as keys across two tables. For full sets of variables, see Tables 2–16.

This research follows best-practice approaches for open and reproducible research planning, execution, reporting, and management (e.g.^41–46^). The experimental design and data collection follows community-approved standards, as detailed below. The data are cleaned and managed using script-based workflows that ensure reproducibility and transparency in R^47^ or MATLAB^48^ (see Table 1 for relevant code). We deposit all data and code in open repositories (e.g., Fig. 2 in^49^). The database consists of 16 main data tables, linked by keys related to time, sampling locations, and species (Fig. 2).

### R packages

We used the R packages dataDownloader^50^, dataDocumentation^51^, data.table^52^, reshape2^53^, readr^54^, rlang^55^, stringr^56^, glue^57^, janitor^58^, lubridate^59^, magrittr^60^, purrr^61^, TNRS^62^, readxl^63^, writexl^64^, googlesheets4^65^, googledrive^66^, baRcodeR^67^, spectrolab^68^, broom^69^, tibble^70^, dplyr^71^, tidyr^72^, tidylog^73^, and tidyverse^74^ for data entry, data wrangling, and data cleaning. The packages ggplot2^75^, ggpubr^76^, ggcorrplot^77^, ggh4x^78^, GGally^79^, cowplot^80^, patchwork^81^, viridis^82^, viridisLite^82^, wesanderson^83^, MetBrewer^84^, RColorBrewer^85^, rcartocolor^86^, pals^87^, gridExtra^88^ and ggridges^89^ were used for visualization. Analyses involved the packages PFTCFunctions^90^, *FAsTeR*^91^, ThermStats^92^ Thermimage^93^, co2fluxtent^94^, RespChamberProc^95^, sarima^96^, cpop^97^, vegan^98^, ggvegan^99^, traitstrap^100^, LeafArea^101^, and performance^102^. Automated setup and processing was carried out using targets^103^, tarchetypes^104^, and usethis^105^ with package installation using devtools^106^ and remotes^107^.

### Research site selection and basic site information

The study area is located in the proposed Witsieshoek Community Conservation Area, in the Witsieshoek component of the QwaQwa Maloti, in the far northern Maloti-Drakensberg Mountain range, South Africa (Fig. 1). The study area is in the Free State province, immediately adjacent to KwaZulu-Natal in the east and the Kingdom of Lesotho to the south, and forms part of Batlokoa Traditional Authority land (customary law/hereditary custodians) under a long-term management lease by Transfrontier Parks Destinations on their behalf for sustainable community development through mountain tourism. The border with KwaZulu-Natal also marks the boundary with the uKhahlamba-Drakensberg Park and UNESCO World Heritage Site.

The study area exhibits strong elevation and topographic partitioning resulting from geological formations and a complex ancient denudational history, as is typical throughout the Maloti-Drakensberg^108^. The lower reaches (up to 1,800–2,000 m) are dominated by sandstone cliffs and valleys of the Clarens Formation, replaced by Drakensberg Basalts to the summit. Topography comprises variations of sheer cliffs staggered by extensive plateaux and spurs, deep cutbacks, valleys, and innumerable waterfalls. The main vertical escarpment varies from a few hundred metres (at the study site being from 2,600–2,900 m a.s.l) to 1 km high (such as on the adjacent Amphitheatre with its uThukhela Falls of 983 m - currently considered the highest waterfall in the world). The summit plateau undulates, representing the pre-rifted Gondwana palaeo-land surface that was later uplifted in the Miocene and Pliocene to the current elevation.

The area falls within the 36,500 km^2^ Drakensberg Mountain Centre of Floristic Endemism^5^, with some 2,800 recorded vascular plant species of which 9% are endemic to the Centre of Floristic Endemism, and many are endemic to the eastern Afromontane and or eastern Great Escarpment^5,6^. The dominant vegetation type of the lower montane area (<2,300 m) is Northern Drakensberg Highland Grassland^109^ with *Leucosidea sericea* woody thicket expansion, while the upper montane (2,300–2,600 m) and sub-alpine (2,600–2,900 m) are dominated by uKhahlamba Basalt Grassland. These grassland types are characterized by a high diversity of perennial tussock grasses, perennial forbs (suffrutexes), and geophytes, notably *Helichrysum* spp., for which the Maloti-Drakensberg is the center of diversity^5^. The alpine zone is dominated by Drakensberg Afroalpine Heathland, characterised by tall tussock grasses (species of *Merxmuellera*, *Festuca*, and *Tenaxia disticha*), and fynbos-like shrubs (*Erica* spp., *Helichrysum trilineatum*, *Passerina montana*). At low elevations most grass species are C_4,_ with a shift to dominance by C_3_ genera above 2,650 m a.s.l.^110^. The climatic treeline is around 2900 m. The mean annual rainfall in the northern Maloti-Drakensberg is 750–1,350 mm, of which 75% occurs between November and March^111^ in the form of convection thunderstorms; heavy precipitation from autumn cut-off lows is typical and mist and fog contribute to moisture availability across all seasons. There is high moisture seasonality, with winters being typically dry, with heavy night frost; the several snowfall events per winter/spring are associated with low pressure systems (cold fronts and cut-off lows) moving north-east across South Africa and Lesotho from the south Atlantic^112^. The mean air temperature on the nearby Sentinel peak (3,165 m.a.sl.) has been measured to 5.8 °C, placing it within the alpine vegetation zone^113^.

### Experimental design

#### Elevation gradient

The study was conducted along a north-extending mountain spur which extends from the base of the Sentinel Peak north-eastwards and continues for 170 km as both the provincial boundary between the Free State and KwaZulu-Natal provinces and an important biogeographic boundary in South Africa^114^. Five study sites were established along the elevation gradient, each separated by approximately 200 m in elevation at c. 2,000, 2,200, 2,400, 2,600, and 2,800 m a.s.l. As aspect is an important driver of moisture availability in southern African mountains, with strong maritime influence on the Maloti-Drakensberg from the Indian Ocean to the east, we sampled both the east-facing (wetter, prevailing windward) and west-facing (drier, lee) aspects of the spur^115^. At each of the five sites we established ten 1.2 × 1.2 m quadrats (hereafter plots; five on west-facing and five on east-facing aspects) arranged along a horizontal transect within each elevation and aspect with at least 1 m distance between adjacent plots. We recorded the coordinates of each plot using a GPS device (accuracy c. 10 m). Potential plot locations along a transect were rejected if they had > 10% rock or bare soil or were too steep or rugged for our sampling methods (see below). The three lower-elevation sites had burned earlier in 2023, and the two lower-elevation sites were also grazed by free-ranging domestic herbivores (sheep, goats) during data collection.

#### Warming experiment

The 3,064 m a.s.l. site is part of the RangeX project, a climate warming and upslope focal plant transplantation experiment replicated across four countries (South Africa, Switzerland, Norway, and China; see^116^) as part of the International Tundra Experiment (ITEX) network^117^. The RangeX experiment was established in October 2021 at the mountain plateau (−28.75497500 S, 28.86698611 E; Fig. 1). For this study, we sampled the native vegetation within 10 1 × 1 m RangeX sampling plots (five warmed, five ambient) where warming is achieved by Open Top chambers (OTCs) with a base diagonal of 2 m and a height of 0.6 m placed over the plot. The RangeX site is facing northeast, and we therefore categorize the site as being on an east-facing aspect for the purpose of this study to allow comparison with the gradient data (see above).

### Species identification, taxonomy, and flora

Species were identified using regional floras^118–120^. To support identification, three of the authors who have extensive knowledge of the regional flora (PCR, ICS, BM) provided a baseline set of plant names and a “quick guide”. These authors also checked all identifications. For difficult species and specimens, we consulted experts on the relevant taxonomic groups and compared voucher specimens to herbarium specimens at the University of Pretoria Herbarium (PRU). Species names were standardized using the TNRS package^121^. Lichens and bryophytes were not collected or identified.

### Dataset i: Plant species composition

Vascular plant community composition was recorded for all sampling plots in December 2023. We visually estimated the percentage cover for each vascular plant species in each plot. Sum of these covers can exceed 100% due to vegetation layering. We recorded species as ‘reproductive’ if aboveground reproductive organs were observed for at least one individual of the species within the plot, otherwise it was recorded as ‘sterile’.

### Dataset ii: Vegetation height and structure

We visually estimated the percentage area of the plot covered by bare soil, rock, moss, and lichen. We only regarded stones large enough to impede plant growth as part of rock cover. If rock, moss, lichen, or bare soil were present but covered less than 1% of the plot, the cover was noted as 0.5%. To estimate the vegetation height of each plot, we measured vegetation height at five points in the plot (one in the center of each quadrant and one in the center of the plot).

### Dataset iii: Aboveground biomass

Biomass samples were collected from all plots along the elevation gradient at the end of the sampling campaign. Three subplots of 20 cm × 20 cm were delineated in each plot (adding up to a total of 0.12 m^2^), two in diagonally opposite corners and one in the center of the plot. All subplot biomass was clipped at ground level and pooled to create one sample per plot. Biomass was sorted into woody and herbaceous fractions, each of which contained both live and (standing) dead biomass. Litter, bryophytes, and lichens were not collected. Biomass samples were dried for at least 72 h at 60 °C, before being weighed.

### Dataset iv: Aboveground traits

We measured 15 vascular plant functional traits, 13 traits selected to represent the plant size and leaf economic spectra^23^ and two isotope ratios to provide further insights into nutrient and water use: plant vegetative height (cm), plant reproductive height (cm), leaf wet mass (g), dry mass (g), leaf area (cm^2^), leaf thickness (mm), specific leaf area (SLA, cm^2^g^−1^), leaf dry matter content (LDMC, g g^−1^), leaf carbon (C, %), leaf phosphorus (P, %), leaf nitrogen (N, %), carbon to nitrogen ratio (C:N), nitrogen to phosphorus ratio (N:P), C^13^ isotope ratio (δ^13^C, ‰), and N^15^ isotope ratio (δ^15^N, ‰). Methods follow the standardized protocols of Pérez-Harguindeguy *et al*.^46^, with adjustments as detailed below.

1. ***Plant selection and vegetative and reproductive height measurements*** - In each plot, we selected three individuals per vascular plant species present for trait measurements. Individuals were selected to be as far from each other as possible to avoid sampling multiple ramets of a single individual, avoiding juvenile or damaged plants whenever possible. For each selected individual, we measured standing vegetative and reproductive (when relevant) height. Vegetative height was measured as the shortest distance between the highest instance of photosynthetic leaf tissue and the ground, without stretching plants or otherwise manipulating the height. We excluded leaves that did not serve the purpose of resource acquisition, such as phyllary bracts, and prioritized non-cauline leaves in vegetative height and leaf sampling (detailed below). We similarly measured reproductive height as the distance between the ground and the individual’s highest reproductive structure (bud, flower, seed), without stretching plants or otherwise manipulating the height.
2. ***Leaf sampling -*** From each selected individual we collected one mature, fully-grown, sun-exposed leaf, avoiding leaves with signs of herbivory or pathogen damage whenever possible. Petioles and stipules were included, when present, and we referenced published floras^118,122,123^ to identify leaf parts for each species. For Poaceae, we sampled the blade only, cutting these at the auricle to avoid sampling sheaths. For rosette/creeping plants and Poaceae, we prioritized basal leaves for collection, if appropriate basal leaves were not available, we used stem leaves. For species with small leaves, we collected multiple leaves equivalent to an area of 3 cm^2^. A subset of the leaves was also analyzed for leaf hyperspectral reflectance (dataset viii). Additionally, traits were also measured on leaves from plants sampled for root traits and leaf Assimilation-temperature response datasets (dataset v and vii) to allow pairing of data. For a few plots at the lower sites, we collected leaves outside the plots (within 1.2 m) to minimize disturbance prior to the ecosystem flux (dataset x) measurements.
3. ***Leaf storage and processing*** - Leaf samples were stored in plastic self-sealing bags with damp paper towels and at ambient temperature until arriving at the field station, where they were stored in the fridge at 4 °C until processing (within 48 h). During the steps described below, the leaves were returned to their self-sealing bags between each step until they were stored in paper envelopes after the leaf thickness measurements. When a sample entered processing, we first verified the species identification. Samples were then trimmed to include one entire leaf, including petioles and stipules when present, but not the sheaths of graminids, per sample. We cut off any stem tissue that pulled off with sessile leaves (e.g., *Craterocapsa tarsodes, Senecio glaberrimus, Helichrysum dasycephalum*). In the case of plants with leaves that extended underground (e.g., some Cyperaceae, Poaceae, bulbiferous forbs), we cut the leaf where it turned green and removed any white tissue at the base. White tissue was not removed for non-bulbiferous forbs such as *Helichrysum*. For small-leaved species where we sampled multiple leaves per sample (see above) we counted the number of leaves in each sample to allow dividing total sample leaf area and mass by the number of leaves to obtain leaf-level data. We used a light brush to remove soil and other detritus from leaves, especially for leaves with short, rough or strigose hairs (e.g., *Helichrysum pallidum*, *Helichrysum nudifoium*).
4. ***Leaf wet mass*** - Fresh leaf samples were patted with a dry paper cloth to reduce excess surface moisture. Each leaf was weighed to the nearest 0.0001 g using a Mettler AE200, Mettler TOLEDO, or AG204 DeltaRange (±0.1 mg).
5. ***Leaf area*** - Leaves were scanned using a custom-programmed Raspberry Pi in combination with a Canon LiDE 220 or 400 flatbed scanner at 300 dpi and full color range, ensuring leaf blades were unfolded, and leaflets, petioles or stipules did not overlap. Leaf area was calculated using ImageJ^124^ and the LeafArea R package^125^. We manually checked leaf scans images from ImageJ to ensure that the full area of the leaf was computed, and when it was not we manually recolored sections of the leaf that had been missed. Grasses with tightly folded leaves (*Rendlia altera, Microchloa caffra, Koeleria capensis, Ficinia cinnamomea, Elionurus muticus)* were scanned folded, and the area multiplied by two to obtain leaf area.
6. ***Leaf thickness*** - We measured leaf thickness using digital calipers at three places along the leaf, avoiding the midrib and major leaf veins. No measurements were taken for the thickness of the petiole or stipule. Grasses with tightly folded leaves were left folded and resulting measurements divided by two.
7. ***Leaf dry mass*** - Leaves were oven dried for >72 h at 65 °C before being weighed to the nearest 0.0001 g.
8. ***Specific leaf area (SLA)*** - was calculated by dividing leaf area by the leaf dry mass.
9. ***Leaf dry matter content (LDMC)*** - was calculated as the leaf dry mass divided by the leaf wet mass.
10. ***Vascular plant leaf stoichiometry and isotopes*** - Three samples per plot and species (n ≈ 900) were selected for chemical analyses. Sample preparations and Leaf P measurements were done at the Department of Ecology and Evolutionary Biology, University of Arizona, USA. Each leaf sample was ground to a uniform powder. Leaf P was determined using persulfate oxidation and the acid molybdate technique^126^, followed by colorimetric measurement of the phosphorus concentration with a spectrophotometer (ThermoScientific Genesys20). Leaf N, C, δ^15^N, and δ^13^C were measured in the Department of Geosciences Environmental Isotope Laboratory at the University of Arizona, USA. These measurements were done using flash combustion analysis of organic matter via a continuous-flow gas-ratio mass spectrometer (Finnigan Delta PlusXL) coupled to an elemental analyzer (Costech), which involved combusting samples of 1.0 ± 0.2 mg in the elemental analyzer. Standardization relied on acetanilide for elemental concentration, NBS-22 and USGS-24 for δ^13^C, and IAEA-N-1 and IAEA-N-2 for δ^15^N. Precision is at least ± 0.2 for δ^15^N (1 s), based on repeated internal standards. These data were used to calculate ratios between C:N and N:P.

### Dataset v: Root traits

Following standardised root trait protocols^127,128^, we measured 10 root functional traits that reflect root size and trade-offs in the roots’ conservation (i.e., resource use and acquisition) and collaboration (i.e., mycorrhizal symbiosis) gradients as well as being indicators of growth rate and environmental tolerance in plants: maximum rooting depth (cm), root wet mass (g), root dry mass (g), total root length (mm), total root volume (cm^3^), root dry matter content (RDMC, mg g^−1^), root tissue density (RTD, g cm^−3^), mean root diameter (RD, mm), specific root length (SRL, total root length/root dry mass, m g^−1^), branching intensity (BI, mm^−1^), aboveground biomass (AGB, g), belowground biomass (BGB, g) and the belowground to aboveground biomass ratio (BGB_AGB, g g^−1^).

Root trait measurements are time-consuming, necessitating a sampling design focusing on a subset of species and sites for this dataset. We selected five focal species (three grasses, *Themeda triandra*, *Eragrostis capensis*, *Harpochloa falx*; and two forbs, *Helichrysum pilosellum* and *Senecio glaberrimus*) as they were present and relatively abundant across the full elevation gradient. These species represent two major growth forms and families (graminoids, Poaceae; forbs, Asteraceae). Roots were sampled from four sites (2,200 to 2,800 m a.s.l.), as the low elevation site (2,000 m a.s.l.) had compacted, clay soils that made root excavations challenging. At each site, we sampled only on western slopes, which all burned in 2023 (outside the gradient plots, to avoid interference with sampling of other datasets), to standardize for time since the most recent fire.

1. ***Root, rooting depth sampling and storage*** - At each site we extracted five individual plants from each focal species by excavating a 5 cm radius to 10 cm depth, sampling outside of the gradient plots to avoid destructive sampling of the plots. After excavation, we measured the maximum rooting depth as the length of the longest root attached to either the grass species’ rhizome or forb stem. Each full plant was stored in a plastic bag at ambient temperature until arriving at the field station and then refrigerated at 4 °C and processed within 12 h^129^.
2. ***Root processing*** - The aboveground parts were cut off and sampled for leaf traits and biomass (see below) and the whole root system was then submerged in water for 30 min before performing a careful suspension and sieving process for at least 30 min or until the remaining soil was cleaned off (Fig. 3a). We then identified and sorted first to third-order roots (hereafter fine roots). Following the morphometric classification, a maximum of five sections of fully attached fine roots were cut^130^ and stored in water. All fine root traits were then measured across these five sections for each individual plant. *Helichrysum pilosellum* and *Senecio glaberrimus* had tuberous roots. We selected a maximum of five tubers per individual and measured wet and dry mass using the same process as for fine roots. We scanned each set of fine roots with a Canon LiDE 220 flatbed scanner with a single light source at 600 dpi and grayscale, unsharp mask, and backlight correction settings. Root scans were computationally processed using RootPainter^131^ to remove shadows and gap fill light roots (Fig. 3b), see also the Data Validation section for additional information. To compute root traits we used RhizoVision Explorer (version 2.0.3)^132^ with the following parameters for the image pre-processing step: analysis mode for broken roots, convert pixel to physical units using a value of 600 DPI, an image thresholding level of 200, and a 1 mm^2^ maximum size to filter non-root objects. For the feature extraction step, we enabled root pruning with a threshold of 5 and used 8 diameter classes every 0.25 mm from 0 to >  = 2 mm. All scans were then batch processed.

**Fig. 3:**
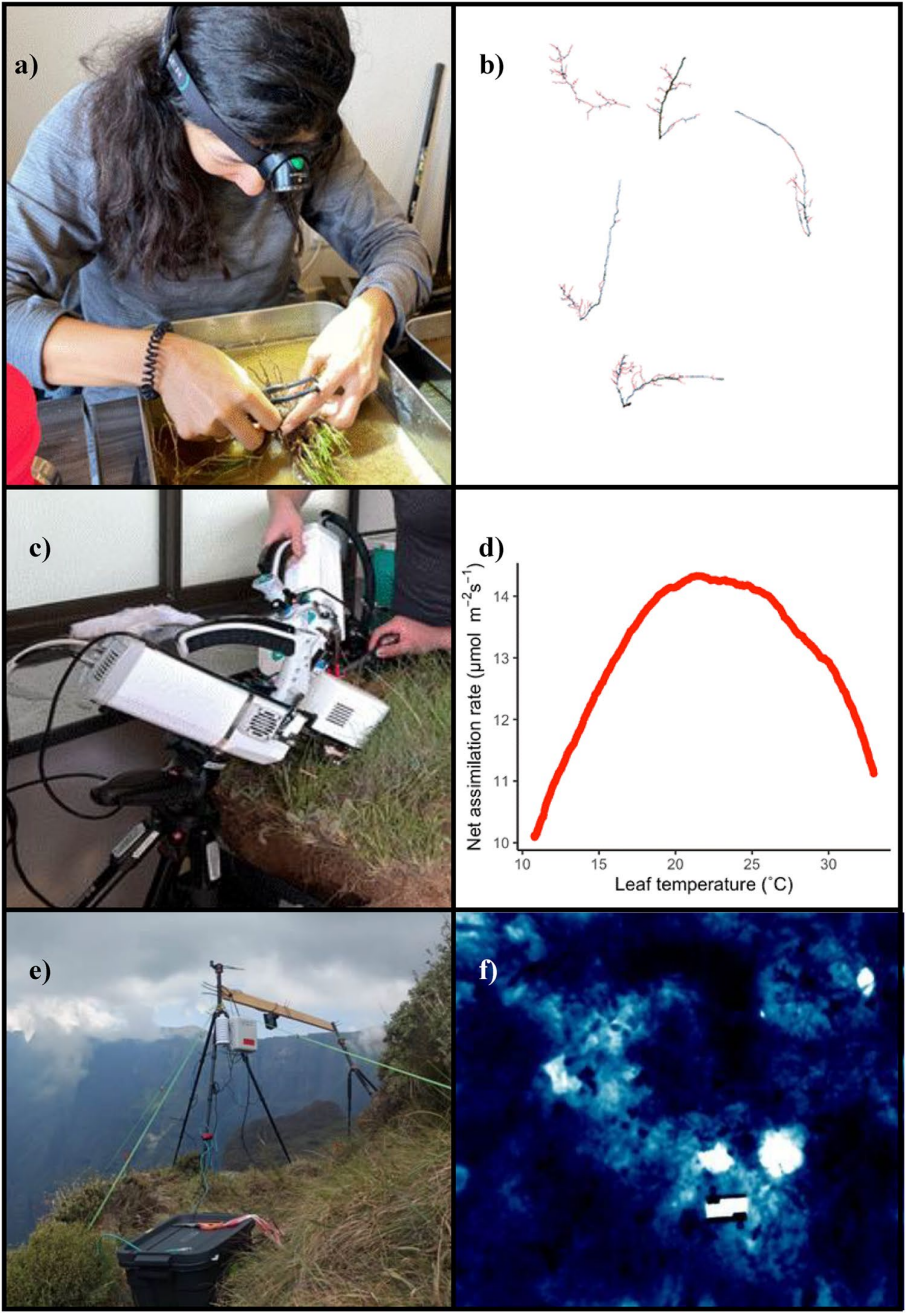
Illustration of the root trait (dataset v), assimilation-temperature response (dataset vii), and vegetation thermal imaging (dataset ix) methods and data. (**a**) Washing the root systems and identifying and dissecting up to five 1^st^ to 3^rd^-order roots per plant to be scanned. (**b**) An example of roots processed through RootPainter^128^ and RhizoVision Explorer (version 2.0.3)^161^ to extract root trait measurements on mean root diameter (diameter classes color coded in the image), total root length, total root volume and branching intensity. (**c**) Collecting assimilation-temperature response data with the LI-COR LI-6800 Portable Photosynthesis System. (**d**) An example assimilation-temperature response curve measures using the *FAsTeR* method^91^. (**e**) FLIR camera field setup for vegetation thermal imaging. (f) An example vegetation thermal image with the calibration plate visible as a white rectangle.
3. ***Aboveground leaf traits and biomass*** - Leaves from each plant were sampled and processed according to the dataset iv procedure (excluding chemical analysis); however, because these plants were not sampled from the plots, the resulting leaf trait measurements are reported in dataset v. The remaining aboveground biomass of each individual was dried for >72 hours at 65 °C before being weighed to the nearest 0.0001 g for each individual.
4. ***Root wet mass*** - The fine root samples were carefully patted dry and weighed to the nearest 0.0001 g.
5. ***Total root length*** - was calculated as the sum of Euclidean distances between the connected pixels in the full root scan.
6. ***Total root volume*** - was calculated as the sum of values from each root pixel by multiplying the cross-sectional area (calculated using the pixel-level root diameter class) by the length of each root pixel in the full root scan, making the assumption that each root is cylindrical.
7. ***Root dry mass*** - The fine root samples were oven dried for >72 h at 65 °C before being weighed to the nearest 0.0001 g.
8. ***Mean root diameter (RD)*** - was calculated as the mean across each pixelized root diameter segment for each diameter class in the root scan.
9. ***Branching intensity (BI)*** - was calculated as the number of branch points divided by the total root length in the root scan.
10. ***Specific root length (SRL)*** - was calculated as the total root length divided by the root dry mass.
11. ***Root tissue density (RTD)*** - was calculated as the root dry mass divided by total root volume.
12. ***Root dry matter content (RDMC)*** - was calculated as the root dry mass divided by the root wet mass.
13. ***Belowground biomass (BGB)*** - The remaining parts of the root system were dried separately for >72 hours at 65 °C before being weighed to the nearest 0.0001 g to determine the belowground biomass of each individual.
14. ***Belowground to aboveground biomass ratio (BGB_AGB)*** - was calculated as the belowground biomass divided by the aboveground biomass.

### Dataset vi: Root biomass mapping

Root biomass profiles were assessed via ground penetrating radar (GPR) using the Tree Radar Unit (TRU™) system (TreeRadar, Inc.) with a 1600-MHz antenna coupled with an odometer wheel for recording distance. We used a hand-push scanner set to 0.5 cm increment recording distance with a soil dielectric constant of 13 and 0–50 cm depth analysis gate. Because vegetation was typically short (<10 cm height), no vegetation was removed before scanning. We did not scan at the highest elevation site (2,800 m) as the terrain was too uneven for reliable results. We measured two GPR-derived variables, amplitude (dB) and pixel count and for root biomass validation we measured root, soil and stone mass (g).

1. ***Root biomass validation (vi-a) -*** To determine if we could build a predictive model for root biomass using the GPR profiles, we tested the correlations between amplitude and pixel count with root biomass (see Data Validation). We scanned two 20 m long transects at least 5 m apart on the western aspects of four sites (2,000, 2,200, 2,400, 2,600 m; Fig. 1) totaling 8 transects. We selected 10 locations at which to sample root biomass. We identified locations by randomly selecting 10 points at least 0.5 m apart and 0–10 cm deep, totaling 80 root biomass samples. To sample root biomass, we removed the aboveground biomass and collected a soil core at approximately 10 cm depth using a 5.7 cm diameter auger. The soil samples were dried at 65 °C for 72 hours, sieved using a 1 mm sieve, and soil, roots and stones were then weighed separately.
2. ***Ground penetrating radar mapping (vi-b) -*** We scanned all plots on both aspects of four sites (2,000, 2,200, 2,400, 2,600 m) through the plot midpoint. Plots were demarcated in the dataset using the built-in GPR odometer marker. These distance values were not always equal to 1.2 m (the plot length) due to uneven terrain.
3. ***Processing and storage*** - All GPR scan profiles were processed using TBA™ root analysis software (v3.9.4) (www.treeradar.com/) to detect root reflection hyperbolas using the auto-detection algorithm, which identifies echo-dynamic patterns that are i) typical of cylindrical objects in shape, such as roots, and ii) exceed the chosen amplitude detection threshold (i.e., sensitivity) in the soil profile. This produces an estimate of the position along the transect (m), depth (cm), amplitude (dB), and the pixel count (integer). The amplitude represents the strength of the return signal at the peak of the reflection hyperbola, while the pixel count represents overall size of the reflection and is suggested to be correlated with root biomass^133^. Together these metrics can be used to infer characteristics of root size, shape and distribution, where pixel count has been shown to correlate with root biomass^133^ and amplitude with root diameter^134^.

### Dataset vii: Leaf assimilation-temperature responses

We measured leaf-level assimilation-temperature response curves for nine focal species, *Dimorphotheca jucunda*, *Eucomis bicolor*, *Helichrysum ecklonis*, *Helichrysum nudifolium*, *Helichrysum pallidum*, *Helichrysum pilosellum*, *Hypoxis costata*, *Senecio glaberrimus*, and *Senecio* cf. *scitus*. Two of the focal species were selected for each site-aspect combination based on their local abundance (dataset i) and sufficient leaf area to fill the LI-6800 chamber. Samples of these species were collected outside plots from the east- and west-facing aspects at the relevant sites along the elevation gradient (Fig. 1). All leaves sampled for the assimilation-temperature response measurements were also measured for aboveground plant functional traits (dataset iv) and leaf hyperspectral reflectance (dataset viii) to allow pairing of data.

#### Field sampling of turfs

We extracted turfs (minimum 25 cm × 25 cm × 15 cm, adjusted to bedrock depth) selected to maximize focal species density and transported them to the laboratory. To minimize disturbance to above- and below-ground organs, the edge of the turf was placed a few centimeters away from focal individuals. Turfs were stored outside in sun-exposed and well-watered conditions until measurements were conducted (within 24 h of turf collection).

#### biLab measurements of assimilation-temperature responses

Assimilation-temperature responses were measured on individual leaves of the focal species within the extracted turfs (Fig. 3c) using three LI-COR LI-6800 Portable Photosynthesis Systems (LI-COR Biosciences Inc., Lincoln, NE, USA).

We first estimated saturating photosynthetic photon flux density (PPFD) for each species using a minimum of three light response curves per species, following^135^. Leaf temperatures were held constant at 20 °C, with LI-6800 flow rate set to 600 µmol s^−1^, relative humidity set to 35%, and reference CO_2_ set to 420 µmol mol^−1^. The PPFD was then decreased in nine steps from 1800 to 10 μmol m^−2^ s^−1^. We estimated the saturating PPFD as 80% of the maximum asymptotic assimilation rate from a fitted light response model^136^. The mean values for each species were: *Dimorphotheca jucunda*, 900 μmol m^−2^ s^−1^*; Eucomis bicolor*, 600 μmol m^−2^ s^−1^*; Helichrysum ecklonis*, 800 μmol m^−2^ s^−1^; *Helichrysum nudifolium*, 700 μmol m^−2^ s^−1^; *Helichrysum pallidum*, 700 μmol m^−2^ s^−1^; *Helichrysum pilosellum*, 600 μmol m^−2^ s^−1^; *Hypoxis costata*, 600 μmol m^−2^ s^−1^; *Senecio glaberrimus*, 600 μmol m^−2^ s^−1^; *Senecio* cf. *scitus*, 1200 μmol m^−2^ s^−1^.

We used the Fast Assimilation-Temperature Response (*FAsTeR*) method^91^ to assess assimilation-temperature responses (Fig. 3d). The youngest fully expanded leaf of each individual was clamped into the LI-6800 chamber, ensuring the chamber area was filled as much as possible. We ensured good contact between leaves and the leaf thermocouple to avoid errors in leaf temperature measurement^137^. The PPFD value was set to the species-specific level previously determined (see above), and relative humidity was held constant at 35%. Leaves remained in the chamber for an acclimation period of at least 20 minutes while the LI-COR Peltier device cooled to the minimum possible temperature, which was dependent on ambient conditions (~5 to 15 °C). Once a stable chamber temperature was reached, we initiated a linear temperature ramp spanning 40 °C. The temperature was ramped at a rate of 1.5 °C min^−1^, for a total time of 30 min post-stabilization. The fan speed was set to 10,000 RPM and chamber flow rates were set to 200–300 μmol m^−2^s. Data were logged every 2 s with 1 s signal averaging over 33 min. In cases where the leaf did not completely fill the chamber, the portion of the leaf that had been enclosed in the chamber was overlaid with a silicone disk and marked with a pen, the area of the marked leaf area measured as described under dataset iv, and assimilation values were recalculated using the measured leaf areas. Post-measurement corrections for IRGA drift and nonequilibrium effects were applied with code from^91^.

### Dataset viii: Leaf hyperspectral reflectance

#### Field sampling and storage

Hyperspectral reflectance measurements were performed on a subset of leaves collected for aboveground trait measurements, excluding leaves that were too small to fill the measurement area of the spectroradiometer (dataset iv), on all leaves collected for leaf assimilation-temperature response measurements (dataset vii), and on all leaves sampled for vegetation thermal imagery (dataset ix), to allow pairing of data. We then selected three species for studies of intraspecific variation, *Senecio glaberrimus, Helichrysum ecklonis* and *Helichrysum pallidum*, which all had relatively large leaves and relatively high abundance at all sites along the elevation gradient and augmented our sampling with *ad hoc* additional samples of these species from outside plots as needed to ensure we had a total number of at least 15 individuals per species, site, and aspect.

All leaves were sampled, stored, and processed following the methods described in dataset iv, except for shorter maximum leaf storage times (see below). This resulted in sampling of leaves for hyperspectral reflectance at both east and west-facing slopes at all five sites along the elevation gradient. This sampling approach allowed for sufficient samples to build robust Partial Least Squares Regression (PLSR) models^138^ for leaf functional traits at the site level, at the species level for our three focal species, and for interspecific, inter-site PLSR models to predict physiological parameters from spectra.

#### Leaf spectroscopy measurements

We obtained leaf-level spectra in the laboratory using an SVC HR1024i spectroradiometer and the LC-RP Pro leaf clip attachment (SpectraVista Corp, Poughkeepsie, NY, USA) within 5 h of sampling and before measuring leaf traits (dataset iv) to avoid damage or desiccation that could alter leaf optical properties. For measurements paired with leaf assimilation-temperature response data (dataset vii), spectra were obtained immediately after gas exchange measurements. Adaxial spectral readings were taken at two to three different locations on each leaf and averaged. Areas with prominent venation or irregularities were avoided. White reference scans were performed every 10 minutes to avoid measurement drift. If needed, the lens was cleaned with a KimTech wipe.

### Dataset ix: Vegetation thermal imagery

*In situ* leaf temperatures and environmental data were recorded at the east and west aspects for the 2,000 and 2,800 m sites, following the methods of^139^. A FLIR A700 thermal infrared imaging camera (Teledyne FLIR, Wilsonville, OR, USA) was mounted at a height of 1.7 m, oriented perpendicular to the ground (Fig. 3e). The camera was controlled by a Raspberry Pi running a Python script adapted from^139^, and captured thermal and RGB images every 5–6 seconds over an 8–10 hour interval (~08:00 to 18:00), covering an area of approximately 1.4 × 1.1 m at a resolution of 640 × 480 pixels. For ground truth calibration, a black reference plate embedded with a type T thermocouple was positioned within the camera frame^129,139^ (Fig. 3f). MATLAB image processing libraries were used to align thermal and RGB images together, and to extract time series of mean surface temperature for vegetation over time^139^. Microenvironmental data were measured at the same frequency as thermal imagery, including humidity and temperature (Atlas Scientific), 10 cm soil temperature (type T thermocouple, Omega Engineering Inc.), and ambient air temperature (bare-wire type T thermocouple, Omega Engineering, Inc.). PPFD (LI-190R quantum sensor, LI-COR Biosciences, Lincoln, NE, USA).

### Dataset x: Ecosystem CO_2_ and H_2_O fluxes

#### Plot-level measurements

We measured ecosystem CO_2_ and H_2_O fluxes at all plots along the elevation gradient (Fig. 1) under daytime and nighttime conditions using a static chamber method, and estimated Net Ecosystem Exchange (NEE), ecosystem respiration (R_eco_), Gross Primary Production (GPP), soil respiration (R_soil_), and water fluxes (ET), following^131^. Day-time measurements capture photosynthetic CO_2_ uptake and respiratory CO_2_ release from the ecosystem (i.e., NEE), whereas nighttime measurements capture R_eco_. We measured R_eco_ in the daytime by covering the chamber with a light-impermeable tarp, and calculated GPP from the daytime NEE and R_eco_ measurements as descibed below.

The chamber, constructed from greenhouse tarp (i.e., permeable for approximately 70% of photosynthetic active radiation (PAR; µmol photons m^−2^ s^−1^, so that net assimilation measurements are likely conservative) with dimensions of 120 × 120 × 120 cm, featured two fans for air circulation and was connected to an infrared gas analyzer (Li-7500, LI-COR Biosciences, Lincoln, NE, USA) to measure CO_2_ and H_2_O fluxes. Air temperature measurements inside the tent were taken on the Li-7500 during the same period as the flux measurements. A flexible draping along the chamber’s base weighed down by a heavy chain minimized air leakage. We used two fans at high speed inside the tent to mix the air.

For each plot, gas fluxes were measured five times: three times in the day and twice at night. The daytime fluxes were taken between 10:00 and 15:00, with each plot being measured 1) under light conditions, 2) in dark conditions (120 sec each), and 3) in ambient conditions (without a tent over the sensor head; 90 sec). Nighttime fluxes were taken between 20:00 and 23:00, with each plot being measured 1) in ambient conditions (90 sec; at every other plot), and 2) inside the light permeable tent (120 sec). These durations mitigate the influence of increasing temperature on the plants within the chamber. To equilibrate air conditions inside the chamber with the ambient air, the chamber was aired for one minute between each measurement.

#### Soil respiration measurements

We measured soil respiration once in all plots between 10:00 and 16:30 using an automated Li-8100A (LI-COR Biosciences, Lincoln, NE, USA). At each plot, we pressed a PVC collar (10 cm diameter) into an area of exposed soil and waited at least 10 min before measuring respiration. Soil collars were inserted to a ~2.5 cm depth, leaving ~5 cm exposed above ground. For each measurement, we closed the chamber for 180 sec, including 120 sec of dead time to allow the air to circulate through the system. The chamber purged for 240 sec between measurements.

#### Calculations

Before using plot level measurements to calculate fluxes, an additional correction was applied to both CO_2_ and H_2_O concentrations to account for the effects of water vapor on infrared light, which introduces error into LI-COR measurements.

1 $${\left[{CO}2\right]}_{{\rm{dry}}}=\frac{1000\times [{CO}2]{wet}}{1000-[H2O]{wet}}$$

2 $${\left[H2O\right]}_{{\rm{dry}}}=\frac{1000\times [H2O]]{wet}}{1000-[H2O]{wet}}$$

where [CO2]_wet_ and [H2O]_wet_ correspond to raw LI-COR outputs.

We calculated NEE from corrected CO_2_ concentration as

3 $${NEE}=\frac{\delta [{CO}2]{dry}}{\delta t}\times \frac{P\times V}{R\times A\times \left(T+273.15\right)}$$

where $\frac{\delta [{CO}{2}]{dry}}{\delta {t}}$ is the slope of the CO_2_ concentration against time (µmol mol^−1^ s^−1^), P is atmospheric pressure (kPa), R is the gas constant (8.314 kPa m^−3^ K^−1^ mol^−1^), T is air temperature measured inside the chamber (°C), V is the chamber volume (m^3^), A is the surface area (m^2^), and 273.15 converts from degrees Celsius to Kelvin.

We used a linear segmentation algorithm to select the best flux time series from the continuously measured CO_2_ and H_2_O values and auxiliary variables to automatically discard data impacted by measurement error and/or noisy events. With the *cpop* package in R^97^, the program detects statistically discrepant linear segments in the data and separates them based on the corresponding time intervals. Simultaneous PAR and signal strength measurements determine whether individual segments should be discarded or kept for flux calculations. The selection criteria are based on thresholds for both PAR and signal strength such that, if the average values for either of the two variables is below a certain threshold for that segment (in this case the thresholds are PAR < 700 *μ*mol s^−1^ and signal strength (SS) < 90%), the data in the segment is discarded. The remaining segments are then used to calculate length-weighted average fluxes. We manually reviewed and adjusted the results of this algorithm as described in Data validation. We report NEE with negative values reflecting CO_2_ uptake in the ecosystem, and positive values reflecting CO_2_ released into the atmosphere.

To account for potential leaks in the tent (e.g., due to windy conditions), we also fitted a nonlinear function in addition to the linear model, following the “leaky chamber approach”^140^ and compared model fit using Akaike information criterion (AIC). We report the estimate of the better fitting model.

We calculated GPP as

4 $${\rm{GPP}}={\rm{NEE}}-{{\rm{R}}}_{{\rm{eco}}}$$

We calculated net primary productivity (NPP) as

5 $${\rm{NPP}}={\rm{GPP}}-({{\rm{R}}}_{{\rm{eco}}}-{{\rm{R}}}_{{\rm{soil}}})$$

where R_soil_ was soil respiration measured from soil collars and R_eco_ was ecosystem respiration measured in a dark tent.

ET was calculated as the change in H_2_O concentration within the closed chamber

6 $${ET}=\frac{\delta [H{2}O]{dry}}{\delta t\,}\times \frac{P\times V}{R\times A\times \left(T+273.15\right)}$$

where $\frac{\delta [H{2}O]{dry}}{\delta {t}}$ is the slope of water vapor concentration over time. We estimated transpiration (T) as

7 $${\rm{T}}={{\rm{ET}}}_{{\rm{light}}}-{{\rm{ET}}}_{{\rm{dark}}}$$

where ET_light_ was ET measured in the uncovered chamber and ET_dark_ was ET measured in the covered chamber. ET_dark_ was assumed to be equivalent to evaporation (E). To check this assumption, we compared ET_dark_ to ET_night_ (where ET was measured at night) and found a considerably lower E at night than daytime measurements, likely because night measurements were taken at a lower temperature than daytime measurements. We also recognize that ET_dark_ may include some transpiration as stomates may not have closed in the time it took us to cover the tent.

From this, we calculated instantaneous carbon-use efficiency (iCUE) as

8 $${\rm{iCUE}}={\rm{NPP}}/{\rm{GPP}}$$

and instantaneous water-use efficiency (iWUE) as

9 $${\rm{iWUE}}={\rm{T}}/{\rm{NPP}}$$

### Dataset xi: Microclimate

#### Elevation gradients

We measured environmental data continuously and during the ecosystem CO_2_ flux measurements. We installed TMS-4 dataloggers (TOMST s.r.o., Prague, Czech Republic) in two out of five plots on each transect from 7 December 2023 to 16 December 2023. At 15 min intervals, the logger measured air temperature at 15 and 2 cm above the soil surface and soil temperature at 6 cm depth (±0.5 °C), and soil moisture at 15 cm depth. Soil moisture was converted from raw electrical signal to soil water content using the silt, sand, and clay data from dataset xii to select the appropriate calibration in^132^. During daytime flux measurements at the 2,400, 2,600, and 2,800 m a.s.l. sites, we continuously measured photosynthetic active radiation inside the flux tent at one second intervals during the entire daytime flux measurement period with a quantum sensor (Li-190, LI-COR Biosciences, Lincoln, NE, USA). At the 2,000 and 2,200 m a.s.l. sites, we noted the PAR values at the beginning and end of our measurements. While ambient flux was being recorded, we measured leaf temperature with a handheld infrared temperature gun (Lasergrip 774, Etekcity, Anaheim, CA, USA) at five points on each plot both day and night. We took four FLIR E-5 Infrared Camera with MSX (FLIR Systems. Wilsonville, OR, USA) images and corresponding natural color images per plot, which made a single composite FLIR image and natural color image per plot at daytime and at nighttime. We extracted per pixel values from all FLIR images and converted to temperature using a modified version of ThermStats::batch_convert^92^. We extracted temperature as recorded by the LI-7500 during ambient flux measurements which were taken at every plot during the daytime (except one plot at 2,600 m a.s.l. East) and every other plot during the nighttime (except 2,400 m a.s.l. West which had no ambient measurements at night and 2,800 m a.s.l.: West which was missing one plot).

#### Warming experiment

Soil moisture and temperatures were measured between 22 January 2022 to 16 September 2024 using six TMS-4 dataloggers in one block comprising six 1m^2^ plots representing the different treatments in the RangeX experiment. Data were processed using the same code as above, and only data from 7 December 2023 to 16 December 2023 in two loggers placed within the native vegetation, one in an ambient temperature and one in a warmed plot, are reported here.

### Dataset xii: Soil texture and nutrients

#### Data collection

Soil depth and soil samples were collected from all plots along the elevation gradient (Fig. 1, Table 1). We collected soil samples from the center of each plot using a soil corer with a diameter of 5.7 cm to a depth of 10 cm, including the organic and mineral soil layer. Soil depth was measured using a 60 cm long probe. Soil samples were collected after all other data from the plots were collected.

#### Processing and storage

The soil samples were air dried at 30 °C for at least one week, then oven dried for two days at 60 °C. Stones and roots were removed using a 2 mm sieve, and samples were analysed for soil texture (sand, silt, clay, and stone %), total nitrogen (N, %), total organic carbon (C, %), total phosphorus (P, mg kg^−1^), soil pH and cation exchange capacity (CEC, cmol kg^−1^) from well-mixed subsamples using dry combustion (N and C)^141^, perchloric acid digestion (P)^142^, 1:2.5 1 M KCl (soil pH) and 0.2 M ammonium acetate (pH 7) (CEC)^143^. All soil analysis was conducted by Bemlab Pty Ltd., Somerset West, South Africa.

### Dataset xiii: Geodiversity and microtopography

#### Data collection

Geodiversity and microtopography data were collected at plot-scale on the elevation gradient in December 2023. Geodiversity variables were documented following^144^, which was developed for mountain environments, as follows: The presence of soil microtopographic features at the soil surface (organic_soil, silt, sand, stone, boulder, rock_outcrop) in each plot was recorded (0/1 scale). The percentage cover of indicators of geomorphological processes (slope_processes, aeolian_processes, fluvial_processes) were visually estimated. Mesotopography was estimated on a 1–10 scale, with 0 reflecting a depression and 10 a ridge. Slope and aspect in degrees were measured using rulers and a compass following^145^.

## Data Records

The data outputs consists of raw and cleaned data files for (i) plant community composition, (ii) vegetation height and structure, (iii) aboveground biomass, (iv) plant functional traits, (v) root traits, (vi) root biomass, (vii) leaf assimilation-temperature responses, (viii) leaf hyperspectral reflectance, (ix) vegetation thermal imagery, (x) ecosystem CO_2_ and H_2_O fluxes, (xi) microclimate, (xii) soil texture and nutrients, and (xiii) geodiversity and microtopography. The final clean data files are available on OSF^146–148^.

### Data organization and structure

All data files are named using the following naming structure: *nr_PFTC7_clean_studysystem_variable_year.csv*, where *nr* refers to the roman dataset number in Table 1; *studysystem* refers to the Elevation Gradient or Warming Experiment (this variable is only used in cases where there are different datasets for the two study systems); *variable* corresponds to the response variable using the terminology in Table 1, and *year* is 2023, the year of data collection. All datasets are structured similarly, sharing some common variables including year, date, site_id, elevation_m_asl, aspect, and plot_id (Table 2), with specific variables that are unique to each dataset (Fig. 2). Note that some datasets share additional variables, such as species and plant_id. Shared variables (bold fonts in Fig. 2) can be used to link different datasets, for example to combine them for analyses.

**Table 2:** Data dictionary with common variables across datasets.

Variable name	Description	Variable type	Variable range or levels	Units	How measured
date	Date the plot was sampled		2023-12-03 - 2023-12-12	yyyy-mm-dd	recorded
aspect	Aspect of the sites, which is either west facing, or east facing	categorical	east, west		measured
site_id	Integer ranging from 1-6 identifying the site	categorical	1 −6		defined
elevation_m_asl	Elevation of site	numeric	2000–3000	m.a.s.l.	measured
plot_id (elevation gradient)	An integer ranging from 0–5 identifying the plot. The numbers 1–5 repeat for each aspect of each site. Plot_id 0 exists only in the dataset viii leaf hyperspectral reflectance, when leaves were collected outside plots.	categorical	0, 1, 2, 3, 4, 5		defined
plot_id (warming experiment)	An integer corresponds to the ID of the RangeX project plot ID	categorical	10.3, 10.4, 5.5, 5.6, 9.5, 9.4, 1.3, 1.4, 6.4, 6.5		defined

Note that there are two variables called plot_id. One is used for datasets from the elevation gradient and the other from the warming experiment.

The code necessary to access the raw data and produce cleaned datasets, along with explanations of the various data cleaning steps, issues, and outcomes, are available in open GitHub repositories, with versioned copies archived in Zenodo (see Table 1). The raw data files are also available at Open Science Framework (OSF) repositories (see Table 1) in a folder called “raw_data”. In this folder there is a separate folder for each dataset containing several raw data files. The folder is named using the roman letter corresponding to Table 1. The Usage Notes section in this paper summarizes the data accuracy and data cleaning procedures, including explanations of and advice on how to deal with various comments and flags in the data, caveats regarding data quality, and our advice on ‘best practice’ data usage. The reader is referred to the code and the detailed coding, data cleaning, and data accuracy comments and the associated raw and cleaned data and metadata tables below for further information. The reader is referred to the code and the detailed coding, data cleaning, and data accuracy comments and the associated raw and cleaned data and metadata tables below for further information.

### Dataset i: Plant species composition

The plant community dataset reports on vascular plant cover, fertility, and associated metadata for 153 taxa in 60 plots (Tables 2, 3). Along the gradient, we registered 153 taxa in 50 plots, with a mean species richness per plot across all sites of 20.8 ± 0.9 species (mean ± SE) ranging from 17.2 ± 0.8 at 2,000 m a.s.l. to 26.6 ± 1.2, at 2,600 m a.s.l.. Species richness was similar on the eastern and western aspects. *Themeda triandra* (Poaceae), *Ficinia cinnamomoea* (Cyperaceae), and *Tenaxia disticha* (Poaceae) were common along the elevation gradient and also had the highest total cover. *Helichrysum* and *Senecio* spp. were the most abundant forbs. The warming experiment had a total of 39 taxa in 10 plots. The mean species richness is 17.4 ± 1.03 and 13.8 ± 2.03 in ambient climate and warmed plots, respectively.

**Table 3:** Data dictionary for the plant species composition (dataset i).

Variable name	Description	Variable type	Variable range or levels	Units	How measured
treatment_warming*	Warming treatment applied; either “ambient” = no treatment, or “warm” = warmed with an OTC	categorical	ambient, warm		defined
treatment_competition*	Competition treatment applied: vegetation = plots containing native vegetation	categorical	vegetation		defined
species	Species name including genus and species	character	*Acalypha punctata* - *Zaluzianskya* sp		identified
cover	Percentage cover of the species per plot	numeric	0.5–80	percentage	recorded
fertility_all	Presence of reproductive structures (flowers, fruits, seeds, buds) present (y) or absent (n)	logical	y, n		recorded

The dataset reports on the abundance of 153 plant species in 60 plots, 153 species in 50 plots on east and west facing aspects in fives sites on an elevation gradient from 2,000–2,800 m a.s.l. and 41 species in 10 plots in an OTC warming experiment at 3,064 m a.s.l, in Witsieshoek, South Africa. Variable names, description, variable type, range or levels, units and short description are given for all variables. Variables indicated with an *, only occur in the warming experiment dataset. Note that variables that are shared across datasets are reported in Table 2.

### Dataset ii: Vegetation height and structure

This dataset reports on the mean height of vegetation and the percentage of the rock covered by bare soil, rock, moss, and lichens in 59 plots along the elevation gradient and in the warming experiment (Tables 2, 4). The mean height of vegetation across all sites was 15.5 cm. Vegetation was tallest at 2,800 m a.s.l. (22 cm) and shortest at 2,000 m a.s.l. (10.9 cm). The cover of moss (<0.5%), lichen (<0.6%), and rocks (<4%) were generally low. The cover of bare soil ranged between 2.5 and 26% and was highest at the lowest site and lowest at 2,800 m a.s.l.

**Table 4:** Data dictionary for vegetation height and structure (dataset ii).

Variable name	Description	Variable type	Variable range or levels	Units	How measured
treatment_warming*	Warming treatment applied; either “ambient” = no treatment, or “warm” = warmed with an OTC.	categorical	ambient, warm		defined
treatment_competition*	Competition treatment applied; vegetation = plots containing native vegetation	categorical	vegetation		defined
variable	Variable describing covers recorded; bare soil, rock, lichen, moss, vegetation height	character	bare_soil_cover rock_cover lichen_cover moss_cover vegetation_height		defined
value	Percentage cover or vegetation height	numeric	0–45	percentage or cm	measured
unit	Units for the variables, where bare soil rock, lichen and moss cover is in percentage and vegetation height in centimeters	character	cm - percentage		defined

The dataset reports on the vegetation of 59 plots at different aspects on an elevation gradient and in a warming experiment in Witsieshoek, South Africa. Variable names, description variable type, range or levels, units and short description is given for all variables. Variables indicated with an *, only occur in the warming experiment dataset. Note that variables that are shared across datasets are reported in Table 2.

### Dataset iii: Aboveground biomass

This dataset contains biomass data from 50 plots along the elevation gradient (all plots along the elevation gradient) and is presented as biomass per 1.44 m^2^ corresponding to the plot size (1.2 m × 1.2 m; Tables 2, 5). Biomass increased with elevation from 210 g at the lowest site to 749 g at 2,800 m a.s.l., with the exception of the eastern side at 2,400 m a.s.l. that had the highest biomass (1,064 g).

**Table 5:** Data dictionary for biomass (dataset iii).

Variable name	Description	Variable type	Variable range or levels	Units	How measured
functional_group	Functional group including woody and herbs	character	herbs - woody		defined
biomass	Biomass value per plot (sum for 3 subplots of 20 × 20 cm)	numeric	2.04–185	g 0.12 m^−2^	measured

The dataset reports on biomass samples in 50 plots at different aspects on an elevation gradient in Witsieshoek, South Africa. Variable names, description variable type, range or levels, units and short description is given for all variables. Note that variables that are shared across datasets are reported in Table 2.

### Dataset iv: Aboveground traits

The majority of the plant functional trait data are reported in two datasets, one for the elevation gradient (dataset iv-a, Tables 2, 6) and one for the warming experiments (dataset iv-b, Tables 2, 6). As plant functional trait data link all the different data and approaches in this study (Table 1), additional leaves were sampled as needed to augment other datasets. Most of these additional leaves are sampled within the gradient sites but outside study plots (Fig. 1) and are given a plot ID of 0 in the gradient trait dataset (Table 6). The exception is leaf traits collated to match the root trait data, which are reported as part of the root trait dataset to facilitate linking aboveground and root traits from individual plants sampled using the root trait protocol (dataset v, see below).

**Table 6:** Data dictionary for the aboveground traits along the election gradient (dataset iv-a and iv-b).

Variable name	Description	Variable type	Variable range or levels	Units	How measured
id	Unique leaf ID consisting of 3 letters and 4 numbers	character	ABU8050 - JED6444		defined
project	Project for which data were collected; TSP = Photosynthesis, TS = Spectroscopy, T = Traits	character	T - TSP		recorded
treatment_warming*	Warming treatment applied; either “ambient” = no treatment, or “warm” = warmed with an OTC.	categorical	ambient, warm		defined
treatment_competition*	Competition treatment applied: vegetation = plots containing native vegetation	categorical	vegetation		defined
plant_id	Individual collection number for each species per plot	numeric	0–3		defined
species	Species name including genus and species	character	*Acalypha punctata* - *Zaluzianskya* sp.		identified
traits	Aboveground traits; veg_height (vegetative_height; cm), rep_height (reproductive height; cm), wet_mass (g), dry_mass (g), leaf_area cm^2^), leaf_thickness (mm), sla (cm^2^g^−1^), and ldmc (g g^−1^)	character	dry_mass_g - wet_mass_g		defined
value	Values for plant functional traits	numeric	0.00006 - 2601	cm, g, mm, cm^2^, cm^2^g^−1^, g g^−1^	measured
unit	Unit for trait values.	character	cm - mm		defined
problem_flag	Flagging potential problems with leaves including damage, missing petioles, and leaflets, etc.	character	damage and missing petiole - missing stipules		recorded

The dataset iv-a reports on 21,921 trait observations of 154 taxa from 50 vegetation plots and adjacent areas sampled across five sites and two aspects along the elevation gradient in Witsieshoek, South Africa. The dataset iv-b reports on 2,494 observations of the covers of 41 taxa in 10 vegetation plots sampled at the 3,064 m a.s.l. site. Variable names, description variable type, range or levels, units and short descriptions are given for all variables. Variables indicated with an * only occur in the warming experiment dataset (dataset iv-b). Note that variables that are shared across datasets are reported in Table 2. Abbreviations: veg_height (vegetative_height), rep_height (reproductive height), sla (specific leaf area), and ldmc (leaf dry matter content).

**Fig. 4:**
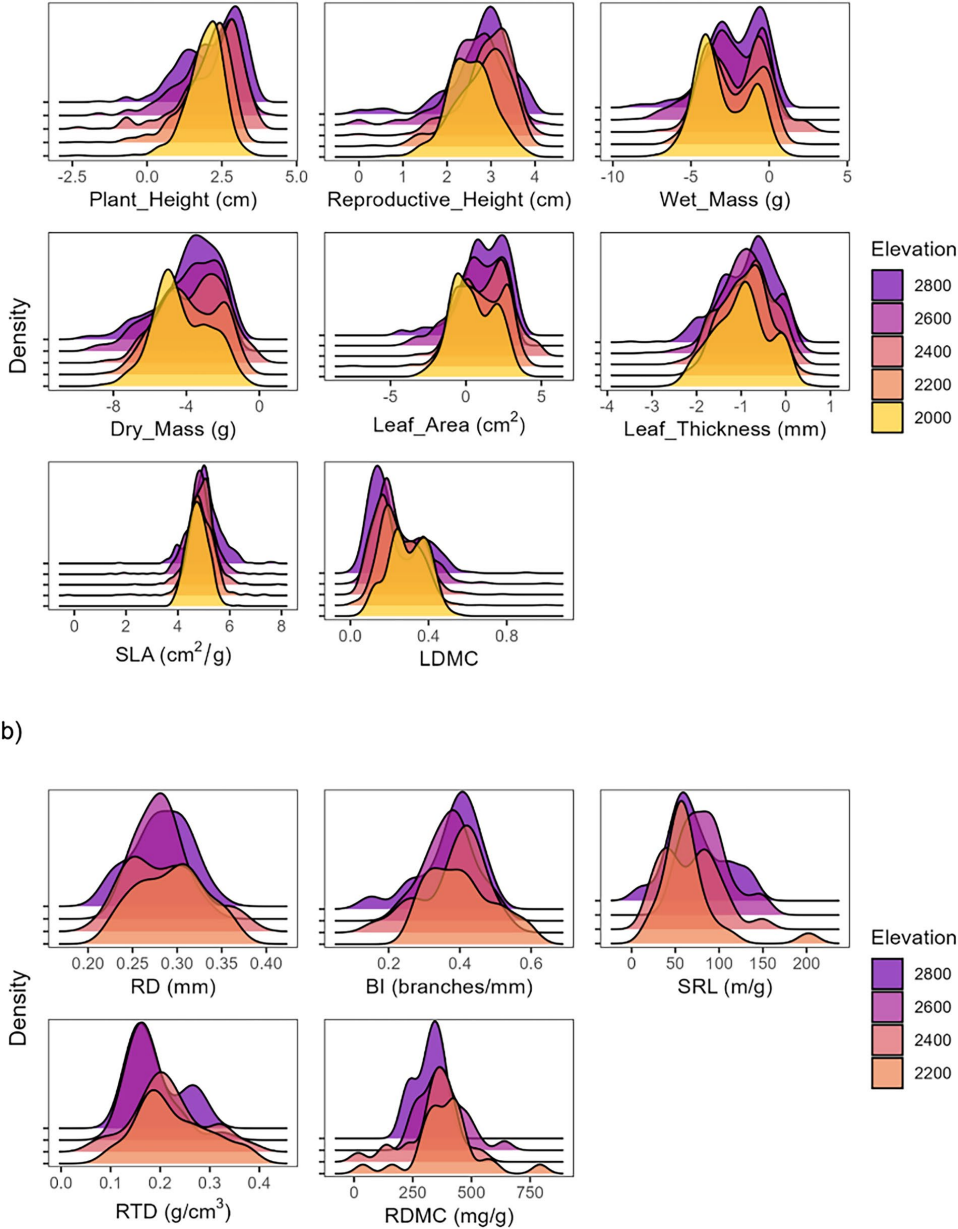
Distributions of trait data along the elevational gradient. Unweighted distributions of trait data (unweighted values) for each site (and aspect) along the 2,000–2,800 m a.s.l in the Maloti-Drakensberg mountains, South Africa. **(a)** The aboveground traits are plant height and reproductive height and leaf wet mass, dry mass, area, thickness, specific leaf area (SLA), and leaf dry matter content (LDMC). The size traits (height, reproductive height, mass, length, area, and thickness) are log-transformed. **(b)** The root traits were sampled at all sites except the lowest elevation. Traits are mean root diameter (RD), branching intensity (BI), specific root length (SRL), root tissue density (RTD), and root dry matter content (RDMC). One value > 0.5 g cm^−3^ for RTD was removed as an outlier for display purposes.

For the elevation gradient, we report on physical and structural traits (plant height, wet and dry mass, leaf area, leaf thickness, specific leaf area [SLA], and leaf dry matter content [LDMC]) measured on 3,038 leaf samples from 156 taxa across all sites and treatments (Table 5, Fig. 4a). Of these, 2,889 leaves were sampled using the plot-level traits protocol, of which 966 were also measured for leaf hyperspectral reflectance (dataset viii). An additional 147 leaves originate from the leaf assimilation-temperature response sampling (dataset vii). Collectively, these leaves resulted in 21,921 trait observations (site × aspect × plot; Table 1). There are a similar number of leaves from both aspects (east: 1,528, west: 1,578), with some variation in leaf numbers across elevation (2,000 m a.s.l.: 622; 2,200 m a.s.l.: 733; 2,400 m a.s.l.: 585; 2,600 m a.s.l.: 680; 2,800 m a.s.l.: 490). For 86% of plots, we have trait measurements for species making up at least 80% of the cumulative cover, making this dataset useful for exploring intraspecific trait variation.

In the warming experiment, we measured physical and structural traits (plant height, wet and dry mass, leaf area, leaf thickness, specific leaf area [SLA], and leaf dry matter content [LDMC]) for 349 leaf samples from 43 taxa across both warming treatments, for a total of 2,494 trait observations (Table 6).

### Dataset v: Root traits

This dataset reports on root and aboveground functional traits of 94 individuals from 5 taxa at the four sites between 2,200–2,800 m a.s.l (Fig. 1, Tables 2, 7). Overall mean RD, BI, SRL, RTD, RDMC (mean ± SD) values are 0.285 ± 0.035 mm, 0.384 ± 0.084 mm^−1^, 73.5 ± 32.2 m g^−1^, and 0.213 ± 0.115 g cm^−3^, and 360 ± 112 mg g^−1^, respectively. RD and BI average values ranged from 0.28 to 0.29 mm and 0.37 to 0.40 mm^−1^, respectively across sites, and did not vary across the elevation gradient, (Fig. 4b). Mean SRL values ranged from 64.08 to 85.43 m g^−1^, with the highest value at 2,600 m a.s.l. RTD values ranged from 0.18 to 0.25 g cm^−3^, with the lowest value at 2,600 m a.s.l. RDMC average values ranging from 326.76 to 391.71 mg g^−1^, with the highest average value at 2200 m a.s.l., (Fig. 4b).

**Table 7:** Data dictionary for the root traits (dataset v).

Variable name	Description	Variable type	Variable range or levels	Units	How measured
id	Unique plant ID consisting of 3 letters and 4 numbers	character	FBC0289 - FGO9191		defined
plant_id	Individual number	numeric	1, 2, 3, 4, 5		recorded
species	Species name including genus and species	character	*Eragrostis capensis, Harpochloa falx, Helichrysum pilosellum, Senecio glaberrimus, Themeda triandra*		identified
traits	Full plant traits including root_depth (cm), no_root_scan (count), root_wet_mass (g), root_dry_mass (g), total_root_length (cm), total_root_volume (mm^3^), rd (mm), bi (count mm^−1^), srl (mg^−1^), rtd (gcm^−3^), rdmc (mg g^−1^), tuber_wet_mass (g), tuber_dry_mass (g), reproductive_height (cm), veg_height (cm), no_leaves (count), leaf_wet_mass (g), leaf_dry_mass (g), leaf_area (cm^2^), sla (cm^2^g^−1^), leaf_thickness (mm), ldmc (g g^−1^), bgb_agb (g g^−1^), aboveground_biomass (g), belowground_biomass (g)	character	root_depth - belowground_biomass		defined
value	Values for full plant traits	numeric	0.0016 – 2471.00	cm, count, g, mm^3^, mm, count mm^−1^, mg^−1^, g cm^−3^, mg g^−1^, cm^2^, cm^2^g^−1^, g g^−1^	measured
unit	Unit for trait values	character	cm - g g^−1^		defined

The dataset contains records for 94 individuals from 5 taxa along four sites at a 2,200–2,800 m elevation gradient in Witsieshoek, South Africa. The table includes variable names, description, variable type, range or levels, units, and measurement type. Note that variables that are shared across datasets are reported in Table 2. Abbreviations: no_root_scan (number of roots scanned), rd (mean root diameter), bi (branching intensity^1^), srl (specific root length), rtd (root tissue density), rdmc (root dry matter content), veg_height (vegetative height), no_length (number of leaves), ldmc (leaf dry matter content), bgb_agb (belowground to aboveground biomass ratio).

### Dataset vi: Root biomass mapping

The root biomass validation dataset (vi-a) reports on the position, depth, amplitude, and pixel count of 80 root detections using GPR matched by root, soil, and stone mass data from 80 soil samples. These 720 measurements were conducted along two transects at each of four sites (2,000, 2,200, 2,400, 2,600 m) on western aspects (dataset vi-a, Fig. 1, Tables 2, 8).

**Table 8:** Data dictionary for the root biomass validation transects (dataset vi-a) using ground penetrating radar and Sampled on an elevation gradient in Witsieshoek, South Africa in 2023.

Variable name	Description	Variable type	Variable range or levels	Units	How measured
transect	Transect number	categorical	1, 2		defined
scan_file	Name of DZT file generated by the GPR	character	ROOT_PFTC7_SITE1_(nogp)_transect_1 - ROOT_PFTC7_SITE4_(nogp)_transect_2		defined
sample_no	Number allocated to soil samples that were taken to determine root density	categorical	1–10		defined
variable	position (m), depth (m), amplitude (dB), pixel_count (count), dry_root_mass (g), stone_mass (g), dry_soil_mass (g), root_to_soil_ratio (g g^−1^), root_to_soil_and_stone_ratio (g g^−1^)	character	position - root_to_soil_and_rock_ratio		defined
value	Value of root biomass mapping and soil collection variables	numeric	0.001243 – 6583	m, dB, count, g, g g^−1^	measured
unit	Unit for variable values	character	m - g g^−1^		defined

Variable names, descriptions, variable types, ranges or levels, units, and short descriptions are given for all variables. Note that variables that are shared across datasets are reported in Table 2.

The ground penetrating radar mapping dataset (vi-b) reports on data from surveying the plots along the elevation gradient for root biomass using the ground penetrating radar (dataset vi-b, Tables 2, 9). We measured the position, depth, amplitude, and pixel count of 3,055 root detections, and we marked the beginning and end of each plot at each site to correlate the plot vegetation data with our root detections.

**Table 9:** Data dictionary for the ground penetrating radar measurements (dataset vi-b).

Variable name	Description	Variable type	Variable range or levels	Units	How measured
scan_file	Name of DZT file generated by the GPR	character	ROOT_PFTC7_SITE1_(nogp)_plot_e -ROOT_PFTC7_SITE4_(nogp)_plot_w		defined
type	The type of measurement that was taken (detect was a direct measurement; marker signals the start and end of a plot)	categorical	detect, marker		recorded
variable	position (m), depth (m), amplitude (dB), pixel_count (count)	character	position - pixel_count		defined
value	Value of root biomass mapping	numeric	0–9902	m, dB, count	measured
unit	Unit for variable values	character	m - count		defined

Sampled on plots at different aspects on an elevation gradient in Witsieshoek, South Africa. Variable names, description, variable type, range or levels, units and short description are given for all variables. Note that variables that are shared across datasets are reported in Table 2.

### Dataset vii: Leaf assimilation-temperature responses

This dataset reports on 127 raw assimilation-temperature curves, resulting in 96 clean curves (e.g., Fig. 3d), for nine species (Fig. 1, Tables 1, 2, 10). During the cleaning process, multimodal curves, curves with poor model fits (*r*^2^ ≤ 0.9), and curves where the fitted optimal temperature was within 3 °C of the minimum or maximum observed leaf temperature were discarded.

**Table 10:** Data dictionary for assimilation-temperature response data (dataset vii).

Variable name	Description	Variable type	Variable range or levels	Units	How measured
obs	Observation	numeric	1–127		recorded
species	Sampled species	character	*Dimorphotheca jucunda - Senecio glaberrimus*		recorded
E	Transpiration rate	numeric	0–0.030	mol m^−2^ s^−1^	derived
A	Photosynthetic rate	numeric	−0.901–26.26	µmol m^−2^ s^−1^	derived
Ca	Ambient CO_2_	numeric	145.584–419.876	µmol mol^−1^	recorded
Ci	Intercellular CO_2_	numeric	−849.256–408.586	µmol mol^−1^	recorded
RHcham	Chamber relative humidity	numeric	1.821–82.752	%	recorded
VPDleaf	Vapor pressure deficit of leaf to air	numeric	0.198–6.144	kPa	derived
gsw	Stomatal conductance rate	numeric	−457.750 −3331.072	mol m^−2^ s^−1^	derived
Flow_s	Sample flow rate	numeric	146.585–681.491	µmol s^−1^	recorded
Flow_r	Reference flow rate	numeric	148.404–867.389	µmol s^−1^	recorded
Txchg	Temperature of the heat exchanger	numeric	−0.717–48.328	°C	recorded

Reports on data from 96 leaves sampled at different aspects on an elevation gradient in Witsieshoek, South Africa names, description, variable type, ranges or levels, units, and short description are given for all variables. Note that variables that are shared across datasets are reported in Table 2.

### Dataset viii: Leaf hyperspectral reflectance

This dataset reports on leaf hyperspectral reflectance for 1,089 leaves from 56 species (34 genera) across all sites (2,000 m a.s.l.: 181,  2,200 m a.s.l.: 263, 2,400 m a.s.l.: _site_214, 2,600 m a.s.l.: 201, 2,800 m a.s.l.: 229) (Fig. 1, Tables 2, 11). This dataset combines three sampling designs. First, all leaf samples from dataset iv that were large enough to fill the measurement area (~1 cm radius) were measured for hyperspectral reflectance prior to functional trait measurements (n = 211) to obtain measurements across as many genera as possible. Second, additional samples were collected for three focal species to allow exploration of variation at the intraspecific level, *Helichrysum ecklonis* (n_total_ = 229), *Helichrysum pallidum* (n_total_ = 234), and *Senecio glaberrimus* (n_total_ = 288). Third, all leaf assimilation-temperature response measurements (dataset vii) were paired with hyperspectral reflectance measurements (n = 127).

**Table 11:** Data dictionary for leaf hyperspectral reflectance (dataset viii).

Variable name	Description	Variable type	Variable range or levels	Units	How measured
id	Unique identifier for each species	character	CXA4387 - JED6444	NA	recorded
plant_id	Replicate measurements per sample	numeric	1–25	NA	recorded
species	Species measured	character	*Acalypha punctata – Zaluzianskya* sp	NA	recorded
‘339.8’- ‘2516.2’	Proportion of light reflected by sample at each wavelength	numeric	0–1	Unitless	measured

### Dataset ix: Vegetation thermal imagery

This dataset reports on a diurnal time series of thermal images from the east and west aspects of the 2,000 and 2,800 m a.s.l. sites, capturing data from sunrise to sunset at each of the four site-aspect combinations (Fig. 1, Tables 2, 12). The camera was positioned to include the focal species from the assimilation-temperature response dataset at each site (dataset vii). Each thermal image is paired with a visual photograph to facilitate region of interest selection. The camera setup includes a black reference plate for temperature calibration, and post-field analysis involves selecting regions of interest for the plants in each image. The time series data contain minor gaps due to battery changes during the measurement periods.

**Table 12:** Data dictionary for vegetation thermal imagery (dataset ix).

Variable name	Description	Variable type	Variable range or levels	Units	How measured
site_id	Site of camera setup	categorical	1–5		recorded
elevation_m_asl	Elevation of site	numeric	2000–2800	m a.s.l.	recorded
aspect	Aspect of site	categorical	east, west		recorded
thermal_mean	Mean leaf temperature for region of interest	numeric	292.03–319.49	K	measured
temp_soil_c	Soil temperature	numeric	281.48–296.57	K	measured
temp_atm	Ambient air temperature	numeric	295.16–309.44	K	measured
temp_black	Reference plate temperature	numeric	283.3–336.8	K	measured
ppfd_mV	Photosynthetic photon flux density sensor raw signal	numeric	0.184–12.47	mV	measured
ppfd_umol_m2_s	Photosynthetic photon flux density	numeric	43.97–2983.97	μmol m^−2^ s^−1^	measured
datetime	Date and time		2023-12-08 11:20 - 2023-12-14 17:55	yyyy-mm-dd hh:mm	recorded

Data measured on two aspects and two sites on an elevation gradient in Witsieshoek, South Africa. Variable names, description, variable type, range or levels, units and short description are given for all variables. Only the major response variables are listed. Note that the raw images are split into different folders, one per site. Note that variables that are shared across datasets are reported in Table 2.

### Dataset x: Ecosystem CO_2_ and H_2_O fluxes

This dataset reports on 194 clean plot-level CO_2_ fluxes (45 NEE, 50 daytime respiration, 49 nighttime respiration and 50 soil respiration measurements) and 198 H_2_O fluxes (50 evapotranspiration, 50 daytime evaporation, 48 nighttime evaporation and 50 soil evaporation measurements) (Fig. 1, Tables 2, 13). From these, we calculated 135 carbon fluxes (45 CUE, 45 GPP, 45 NPP) and 145 water fluxes (50 daytime evaporation, 50 transpiration, and 45 WUE) (Table 14). Note that these data are not standardized for biomass or temperature.

**Table 13:** Data dictionary for ecosystem fluxes (dataset x).

Variable name	Description	Variable type	Variable range or levels	Units	How measured
date_time	Date and time of observation		2023-12-05T08:45:15Z - 2023-12-16T21:00:27Z	yyyy-mm-dd hh:mm:ss	defined
unique_location_id	Concatenated site_id, elevation, aspect, and plot_id	character	2000_east_1 - 2800_west_5		defined
day_night	Measurement performed at daytime or nighttime	categorical	day - night		defined
flux_type	Flux measured or calculated, including cue, evap_day,evap_night, evapotrans, gpp, nee, npp, resp_day, resp_night, soil_evap, soil_resp, transpiration, wue	character	cue - wue		defined
clean_flux_type	Full name and units of the flux	character	Carbon Use Efficiency (GPP/NPP) - Water Use Efficiency (Transpiration/NPP)		defined
flux_value	Flux readings	numeric	−17.879–28.546	µmolm^−2^ s^−1^ (carbon fluxes) or mmolm^−2^ s^−1^ (water fluxes)	recorded
flux_category	Carbon or water fluxes	categorical	Carbon - Water		defined
r_squared	Model fit	numeric	0–0.999		recorded
device	What equipment was used to measure the microclimate value	character	calculated - LI-8100		defined
flag	Quality flag	character	manual_flux_time_selection - okay		defined

Ecosystem CO_2_ and H_2_O fluxes at different aspects on an elevation gradient in Witsieshoek, South Africa. Variable names, description, variable type, range or levels, units and short description are given for all variables. Note that variables that are shared across datasets are reported in Table 2. Abbreviations used in the flux_type column: cue (carbon-use efficiency), evap_day and evap_night (evaporation at day and night respectively), evapotrans (evapotranspiration), gpp (gross primary productivity), nee (net ecosystem exchange), npp (net primary productivity), resp_day and resp_night (ecosystem respiration and day and night respectively), soil_evap (soil evaporation), soil_resp (soil respiration), transpiration (ecosystem transpiration), wue (water-use efficiency).

**Table 14:** Data dictionary of the microclimate and habitat data (dataset xi-a, dataset xi-b).

Variable name	Description	Variable type	Variable range or levels	Units	How measured
date_time	Date and time of observation		2023-12-05 - 2023-12-16 2023-12-05 00:00:00 - 2023-12-16 23:50:00	yyyy-mm-dd hh:mm:ss	recorded
treatment_warming*	Warming treatment applied; either “ambient” = no treatment, or “warm” = warmed with an OTC.	categorical	ambient, warm		defined
treatment_competition*	Competition treatment applied: vegetation = plots containing native vegetation	categorical	vegetation		defined
day_night	Measurement performed at daytime or nighttime	categorical	Day - Night		recorded
climate_variable	Microclimate variables including moisture_soil, temperature_air, temperature_ground, temperature_leaf, temperature_near_surface, and temperature_soil	character	moisture_soil - temperature_soil		defined
value	Temperature or moisture reading discarding values later flagged as suspect	numeric	0.029–72.944	°C for temperature, (m3 water × m−3 soil) × 100 for moisture	recorded
device	Equipment was used to measure the microclimate	character	FLIR - Tomst		defined

Collected at different aspects on an elevation gradient in Witsieshoek, South Africa. Variable names, description, variable type, range or levels, units and short description are given for all variables. Variables indicated with an *, only occur in the warming experiment dataset (dataset iv-b). Note that variables that are shared across datasets are reported in Table 2.

### Dataset xi: Microclimate

The microclimate data contains measurements of air temperature (32,634 observations), near surface air temperature (25,220), ground temperature (3,912,235), soil temperature (25,220), and soil moisture (25,220) for a total of 4,020,529 observations (Fig. 1, Tables 2, 14).

### Dataset xii: Soil texture and nutrients

The soil texture and chemical dataset has 400 observations of 8 soil variables from all plots along the elevation gradient (Fig. 1, Tables 2, 15). CEC had six missing observations, due to insufficient soil remaining for these tests.

**Table 15:** Data dictionary for the soil texture and chemical analysis (dataset xii).

Variable name	Description	Variable type	Variable range or levels	Units	How measured
id	Unique soil collection ID consisting of 3 letters and 4 numbers	character	FGT3613 - FIB5031		defined
variable	Soil texture and nutrient variables including tc_perc (total C; %), tc_N_perc (total N; %), tp_mg_mk (total P; mg kg^−1^), cec_cmol_kg (cmol kg^−1^), pH, clay_perc (%), sand_perc (%), silt_perc (%)	character	tc - silt	—	defined
value	Values for soil texture and nutrients.	numeric	0.16–290	%, mg kg^−1^, cmol kg^−1^	measured
unit	Unit for values	character	% - mg kg^−1^		defined

The dataset contains records for 50 samples from 5 plots on each aspect along five sites across the 2,000–2,800 m elevation gradient in Witsieshoek, South Africa. The table includes variable names, descriptions, variable types, ranges or levels, units, and measurement types. Abbreviations: tc_perc (total C), tc_perc (total N) and tp_mg_mk (total P). Note that variables that are shared across datasets are reported in Table 2.

### Dataset xiii: Geodiversity and microtopography

The geodiversity and microtopography data contains 550 observations of 12 geodiversity and microtopography variables recorded from all plots along the elevation gradient (Fig. 1, Tables 2, 16).

**Table 16:** Data dictionary for the geodiversity and microtopography (dataset xiii).

Variable name	Description	Variable type	Variable range or levels	Units	How measured
variable	Soil geodiversity and microtopography variables including slope (degree), aspect (degree), organic_soil (1/0), silt (1/0), sand (1/0), stone (1/0), boulder (1/0), rock_outcrop (1/0), slope_processes (%), aeolian_processes (%), fluvial_processes (%), mesotopography (0–10)	character	aspect - stone	—	defined
value	Values for soil geodiversity and microtopography	numeric	0–325	degree, percentage, scaled	estimated, measured
unit	Unit for geodiversity and microtopography data	character	degree - percentage		defined

The dataset contains records for 550 samples from 50 plots on each aspect along five sites across the 2,000–2,800 m elevation gradient in Witsieshoek, South Africa. The table includes variable names, descriptions, variable types, ranges or levels, units, and measurement types. Note that variables that are shared across datasets are reported in Table 2.

## Technical Validation

### Community data validation (dataset i)

The data were checked for unrealistic cover values (i.e., individual species with cover values <0.5% or >100%). Due to the lack of a complete and detailed flora for the region, some taxa could not be identified to species-level (e.g., species in the genus *Thesium*), resulting in 23 unidentified species, of which 13 are identified to the genus level, 5 to the family level, and 5 are identified with morphonames. The taxa not identified to species level were all relatively uncommon, comprising a total of 1.1% of the vegetation cover. Trait data were available for 16 of these taxa, comprising 1.9% of trait records. For taxonomic validation see the Methods section.

### Trait data validation (datasets iv and v)

The trait dataset was checked, validated and flagged using the following steps: (i) Duplicates were removed. (ii) Missing or erroneous sample identifications in one or more measurements were checked against field notes and notes on the leaf envelopes. (iii) Unrealistic trait values were checked and corrected against the lab and field notes for typing errors as well as leaf scans were checked for problems during the scanning process (e.g., empty scans, double scans, blank areas within the leaf perimeter, dirt, or other non-leaf objects on scans). All errors that could be resolved by these checks were corrected (e.g., recalculating the leaf area manually for missing leaf parts on the scan, the wrong match between scan and leaf ID, etc.). (iv) Remaining unrealistic trait values were removed from the dataset. This was done for leaves with clearly erroneous trait values such as very high leaf area values, leaf dry matter values higher than 1 g g^−1^, and leaves with specific leaf area values greater than 500 cm^2^/g (see the code^149^ for details). (v) The data were then visualized (e.g., wet mass vs. dry mass) to check for outliers. (vi) For issues that could not be resolved, such as missing and/or damaged leaves, petioles, leaflets and stipules, overlapping leaflets, or juvenile plants leading to potential issues with specific trait values, we added a column problem_flag describing the issues with the data when relevant for a specific leaf and trait.

### Root traits data validation (dataset v)

We tested computational and manual image processing methods to correct root shadows and, in some cases, poor backlighting causing faint, light roots in the root scans.

For computational image processing, RootPainter^128^ was used as a trained deep-learning model (convolutional neural network) to segment roots in the scan images. After masking out image borders and noise (e.g., barcodes) using GIMP (www.gimp.org/), we generated a training dataset of 5 tiles per image with 800 pixels and omitted any tiles absent of roots, producing 135 tiles. Following the methodology by Smith *et al*.^128^, 6 tiles were trained before producing a predictive model. We then manually annotated 101 tiles by correctively specifying root (foreground) and non-root (background) pixels in the user interface. If new tiles produced a sufficiently improved DICE score, RootPainter produced an updated model. After 101 tiles, the DICE score stabilized at > 0.95, suggesting a strong capability of the model to distinguish roots from the background and/or shadows. The model was then used to segment all roots across the original scan images.

For manual image processing, we used GIMP to erase the shadows by using the eraser tool and following the root borders of each root section for 20 scans. Additionally, we repainted some of the roots using the brush tool where they were faint. We set the brush color to black and its size to a similar or slightly narrower width of the root we were painting. We cleaned at least five images per species following this protocol to assess the accuracy of the computational image processing. Raw and image-processed scans were then processed using RhizoVision Explorer as described in Methods Dataset (v) above.

Paired samples t-tests were performed to test for similarities in measured root trait values derived from raw and image-processed scans. The traits used for these comparisons were the total root length, mean root diameter, total root volume and branching intensity. Normal distribution of the difference in functional trait values between the methods used was tested using a Shapiro-Wilk test. If the difference was not normal after using log and sqrt transformations, a Wilcoxon Rank sum test was used instead of the parametric paired samples t-test.

When comparing raw scans against scans edited using RootPainter (n = 94 individuals), we found that in the raw scans total root length, total root volume and branching intensity are significantly overestimated with their respective mean values 22.9, 82.3, and 401.8% higher (total root length: t_93_ = 4.52, p = 1.78e^−5^, total root volume: t_93_ = 6.79, p = 1.04e^−9^, branching intensity: t_93_ = 58, p = 8.17e^−75^), while mean root diameter showed no significant difference between these methods (t_93_ = 0.44, p = 0.66). Similarly, when comparing raw scans against the 20 individuals edited in GIMP, we found that in the raw scans mean root diameter, total root volume and branching intensity were significantly overestimated with their respective mean values 18.1, 79.8, and 60.4% higher (mean root diameter: t_19_ = 8.87, p = 3.53e^−8^, total root volume: t_19_ = 4.55, p = 2.21e^−4^, branching intensity: t_19_ = 9.52, p = 1.16e^−8^), with no significant difference between total root volume (t_19_ = 0.59, p = 0.562). Scans edited using GIMP provided significantly higher values than those edited using RootPainter for total root length and branching intensity, and lower values for mean root diameter (total root length: t_19_ = −2.56, p = 1.93e^−2^, mean root diameter: t_19_ = 9.01, p = 2.74e^−8^, branching intensity: t_19_ = −12.6, p = 1.09e^−10^), however, total root volume showed no difference between these methods (t_19_ = 1.13, p = 0.27).

Overall, these results provided us with confidence that the derived root trait values calculated from RootPainter^128^ are the most accurate given that the presence of shadows and different colorations in raw scans result in an overestimation of trait values; while the manual cleaning process with GIMP impacted the mean root diameter calculation. Moreover, RootPainter is a time effective and efficient tool that allowed us to rapidly process 90 + scans, whereas manually editing scans using GIMP is extremely time consuming and prone to user errors (~3 hours of cleaning per scan).

### Root biomass mapping data validation (dataset vi)

We compared the root-to-soil ratio (g g^−1^) with the amplitude and pixel count data to verify if GPR data can be used to estimate root biomass. We expected the root-to-soil ratio to increase with the increase in pixel count and amplitude. The relationship was assessed with a linear model using R package lme4^150^. We did not find a significant relationship between root density and the pixel count (adj-R^2^ = 0.01, t = −1.44, p = 0.15) nor amplitude (adj-R^2^ = −0.01, t = −0.35, p = 0.73), suggesting that the GPR was not able to accurately measure root biomass in this grassland ecosystem.

### Assimilation-temperature response data validation (dataset vii)

Each assimilation-temperature response curve was assessed for unimodality and curves with multiple peaks or abnormal fluctuations due to error in the chamber controls were discarded. Multiple curves were discarded because they did not include the ramp up to the peak or the ramp down from the peak due to insufficient temperature range in the chamber.

### Leaf hyperspectral reflectance validation (dataset viii)

Spectral curves were immediately checked for irregularities and documented in a metadata file. Some leaves were excluded from scanning because they were too small or damaged.

### Vegetation thermal imagery data validation (dataset ix)

Images before 08:30 h were discarded due to errors while starting up the system. Corrections were made during image processing to correct any field errors noted such as missing sensors.

### Ecosystem CO_2_ flux validation (dataset x)

After the first round of running our flux calculating scripts, we visually assessed each measurement for fit and quality on a plot of CO_2_ or H_2_O concentration vs. time. Where the code had selected a non-optimal time of the flux measurement, we manually input a new time that was visually consistent with a good flux measurement. We discarded fluxes that changed over time in a way inconsistent with biology (e.g., decreasing CO_2_ concentration at night). When the break point function found multiple fluxes within a single measurement, we kept the flux with the lower AIC.

### Microclimate data validation (dataset xi)

We visualized the microclimate data and removed unrealistic high or low values (n = 7,879). Data from automated loggers was trimmed to time points after the loggers had been installed and acclimated. We removed erroneous values based on field notes (e.g., IR temperature records made on bare soil, n = 19).

### Soil texture and nutrients validation (dataset xii)

We visualized the soil data for outliers and subsequently included all observations.

### Geodiversity and microtopography validation (dataset xiii)

Data from the 2,400 m a.s.l site is missing .

## Usage Notes

### Additional resources

The trait measurement protocols, data management, and data science methods used for the PFTC7 course and data campaign are further described in^151^.

### Data use and best practice notes

The data are provided under a CC-BY license. We suggest that data presented here and accessed through the OSF repositories, including future additions to the chemical trait data, be cited to this data paper. We appreciate being contacted for advice or collaboration, if relevant, by users of these data.

### Field work notes

These data were collected at peak growing season. The short-term fire history and grazing intensity varied across the study sites, with the consequence that some plots had been more recently burned, and/or experienced higher grazing intensity than others. The 2,000 m a.s.l. site was heavily grazed (mainly by cattle). The site at 2,200 m a.s.l. had burnt in the previous winter and showed signs of light grazing. The western aspect of the site at 2,400 m a.s.l had burnt in the previous winter, but the eastern aspect did not. The site showed little signs of grazing. The sites at 2,600 and 2,800 m a.s.l. did not burn in the previous winter and showed little signs of grazing. The rainfall for November and December 2023 (384 mm) was slightly higher than average (mean ± SD total rainfall for November and December = 316 ± 117 mm, data from c. 40 km south-east of Witsieshoek at Cathedral Peak at 1,860 m a.s.l for 2012–2023)^152^.

### Community composition (dataset i)

A full species list is provided in the data repository, showing which taxa occur in the plant community and/or aboveground trait data. Fertility values are missing where we forgot to note the fertility status of a species. Identifications for some species that were sampled before or after their flowering seasons are tentative, but made by local experts (e.g., *Satyrium longicauda*).

### Vegetation height and structure (datasets ii, iii)

Plot 2 at the eastern aspect of the site at 2400 m a.s.l. is missing bare soil, rock, lichen and bryophyte cover.

#### Aboveground traits (datasets iv and v)

Potential issues with trait are flagged in the data. The user should decide to include or exclude these trait observations. Chemical trait data are in progress and will hopefully be ready before the data paper is published.

#### Root traits (dataset v)

Individual plants collected for the below-ground traits were collected away from the established plots used in the rest of the study as the collection of plants for this purpose would have been too destructive. We only collected plants to a depth of 10 cm; therefore the maximum rooting depth is likely an underestimate for our samples.

#### Root biomass mapping (datasets vi-a, vi-b)

We used a dielectric constant value of 13 as the soils were determined to be average in soil moisture; this value can be adjusted in post-processing using the DZT files within the TBA software. The GPR was used on rocky, undulating terrain. Care was taken to avoid losing contact between the GPR and the ground surface; however, we were not able to quantify how this may impact the GPR readings.

#### Leaf assimilation-temperature response (dataset vii)

A variety of thermal performance curves can be fit to the data with the rTPC package^153^.

#### Vegetation thermal imagery (dataset ix)

The Atlas air temperature sensor stopped functioning at the 2,000 m a.s.l. east site, which might challenge comparing canopy-level vegetation responses across sites.

#### Ecosystem CO_2_ and H_2_O fluxes (dataset x)

Ecosystem CO_2_ and H_2_O fluxes were initially measured at the 2,000 m a.s.l. site prior to a storm system that developed several days into the course (following a long dry period). As storms can significantly impact soil moisture and thus CO_2_ and H_2_O fluxes, this site was re-measured after the storm system, along with the other sites to allow comparisons across the elevation gradient. The original ecosystem flux measurements before the storm are available in the raw data on OSF^146^, but were not included in the clean data files.

The temperature sensor of the LI-COR 8100 (used to measure soil respiration) was malfunctioning and the temperature values were omitted from the clean data. These measurements can still be found in the “raw_data” folder in this database.

No ambient measurements were taken at the western aspect of the 2400 m a.s.l. site at night.

#### Microclimate (dataset xi)

Note that the microclimate was recorded over a limited period of time and has limitations in reflecting the growing season microclimate at these sites.

Handheld FLIR camera images for each plot were captured immediately after flux measurements for most plots. The images from the 2,000 and 2,200 m a.s.l. sites (daytime) and 2,000 m a.s.l. (nighttime) were overwritten due to the very limited amount of sampling time at the 3,064 m a.s.l. site. FLIR images from these sites were taken at a comparable time 8-9 days after flux measurements for these plots. At 2,600 m a.s.l. site plots 1, 2, and 5 west daytime were overwritten and not replaced. The 3,064 m a.s.l. site was only visited in the daytime, so it has no corresponding nighttime data. LI-7500 temperatures are missing from one plot at 2,600 m a.s.l. east (daytime), 2.400 m a.s.l. west (at night), and one plot at 2,800 m a.s.l west (daytime).

#### Soil texture and nutrients (dataset xii)

There was insufficient mass to analyze CEC values for five samples.

## Data Availability

The code used for checking, cleaning, and analyzing the data, is available in open GitHub repositories^147,149,154–157^, of which versioned copies are available at Zenodo.
